# Conservation and divergence of ciprofloxacin persister survival mechanisms between *Pseudomonas aeruginosa* and *Escherichia coli*

**DOI:** 10.1371/journal.pgen.1011840

**Published:** 2025-09-02

**Authors:** Gabrielle Leon, Mark P. Brynildsen

**Affiliations:** 1 Department of Chemical and Biological Engineering, Princeton University, Princeton, New Jersey, United States of America; 2 Omenn-Darling Bioengineering Institute, Princeton University, Princeton, New Jersey, United States of America; University of Texas McGovern Medical School: The University of Texas Health Science Center at Houston John P and Katherine G McGovern Medical School, UNITED STATES OF AMERICA

## Abstract

Studies have shown that DNA damage repair systems, including homologous recombination (HR) and the SOS response, are important for fluoroquinolone (FQ) persistence of *Escherichia coli*, which has been the workhorse organism of persister research. We sought to explore whether those systems are also important for FQ persistence of *Pseudomonas aeruginosa*, a common cause of lung infections in cystic fibrosis patients, which can be treated with FQs such as ciprofloxacin (CIP). Notably, *P. aeruginosa* has important differences in its DNA damage repair capabilities compared to *E. coli* that include the machinery needed to conduct non-homologous end-joining (NHEJ), Ku and LigD. Using a genetic approach, we found that loss of HR significantly depressed persister levels of *P. aeruginosa* to CIP during stationary-phase, but not in exponential-phase. This differed from *E. coli* grown in identical conditions, where loss of HR reduced survival in both stationary- and exponential-phase populations. Similarly, an inability to induce the SOS response reduced survival during both growth phases for *E. coli* but only in stationary-phase for *P. aeruginosa*. Loss of NHEJ machinery in *P. aeruginosa* did not impact persister levels during stationary- or exponential-phase, whereas overexpression of NHEJ machinery in *P. aeruginosa* had toxic effects. In addition, the generality of findings to another FQ, levofloxacin, and a recent clinical isolate, MRSN 1612, were confirmed. These results demonstrate that HR and the SOS response are important to CIP persistence of stationary-phase *P. aeruginosa*, dispensable to CIP persisters in growing *P. aeruginosa* cultures, and that the contributions of systems to *E. coli* persistence do not directly translate to persisters of *P. aeruginosa*.

## Author summary

Small subpopulations of bacteria can transiently exhibit high tolerance to antibiotics, and those bacteria are called persisters. Most persister research has been conducted with *E. coli*, with the assumption that observations made will translate to other bacterial species. Here we examined how well knowledge of persistence gained with *E. coli* translates to the dangerous Gram-negative pathogen *P. aeruginosa* when the antibiotics used are fluoroquinolones, whose well-known members include ciprofloxacin and levofloxacin. We observed examples of conservation and divergence in persistence mechanisms between *P. aeruginosa* and *E. coli* with respect to the DNA damage repair pathways that are important for persisters to survive ciprofloxacin treatment. In addition, we demonstrated that mechanisms important for ciprofloxacin persistence were also important for levofloxacin persistence in *P. aeruginosa*, and that the impact of loss of homologous recombination on ciprofloxacin persistence was conserved between distinct strains of *P. aeruginosa*, with one being a recent clinical isolate. These results demonstrate that persistence mechanisms between *P. aeruginosa* and *E. coli* can have shared and distinct features, which illustrates the importance of extending persister studies to species beyond *E. coli*.

## Introduction

Bacterial persistence describes a phenomenon where subpopulations of bacteria are killed more slowly during antibiotic treatment than the majority of the population [1,2]. Persisters, the cells that are killed more slowly, differ from resistant cells because they do not exhibit increased minimum inhibitory concentrations (MICs), and when the antibiotic stress is removed and persisters produce a new population, the new culture exhibits the same killing dynamics as the original culture [1,2]. Persistence has been observed in many bacterial species [3–9], including *Escherichia coli* and the ESKAPE pathogen *Pseudomonas aeruginosa*, the latter of which is a common cause of nosocomial infections and infections in immunocompromised individuals, including lung infections in cystic fibrosis patients, burn wound infections, urinary tract infections, and keratitis [10–12]. Indeed, persisters have been identified in clinical *P. aeruginosa* isolates taken from cystic fibrosis patients [3,13,14]. Mulcahy and colleagues found higher persister levels in late *P. aeruginosa* clinical isolates compared to early isolates in 10 of 14 patients examined, suggesting persistence may contribute to recurring *P. aeruginosa* infections [13]. More recently, Bartell and colleagues found that 19% of 460 *P. aeruginosa* clinical isolates taken from 39 cystic fibrosis patients’ airways exhibited a high-persister phenotype to the fluoroquinolone (FQ) ciprofloxacin (CIP), and their study suggested that persistence contributed to antibiotic treatment failure [3]. Beyond their slow death rates complicating antibiotic treatments and leading to relapse infections, studies have shown that persistence can foster the development of antibiotic resistance [15–17]. Therefore, increased understanding of persistence has the potential to reduce incidences of antibiotic treatment failure and resistance development.

*P. aeruginosa* infections can be treated with a variety of antibiotics, including the FQs CIP and levofloxacin (LEV) [18,19]. FQs kill both growing and nongrowing bacteria by causing DNA damage through targeting of DNA gyrase and topoisomerase IV [20–23]. When bacteria experience DNA damage, they often respond to that damage by activating the SOS response, which occurs through RecA induced-autocleavage of the transcriptional repressor LexA [24,25]. Specifically, the protein RecA binds to single-stranded DNA and filaments along those strands [24,25]. Nucleofilaments of RecA bound to single-stranded DNA interact with LexA, which is bound to DNA as a dimer, and catalyze its autocleavage and release from SOS boxes that results in derepression of SOS genes [24,25]. The SOS regulon includes genes encoding for a variety of functions, with many associated with DNA repair such as *recA*, *recN*, and *dinG* [24–28]. As part of their lethal activity, FQs can cause DNA double-stranded breaks (DSBs) [21,29–31] that bacteria can repair with homologous recombination (HR), which involves RecA, the exonuclease RecBCD, and an intact template strand of DNA [32–34]; or non-homologous end joining (NHEJ), which involves Ku and LigD and does not require an intact template strand of DNA [35,36]. Importantly, not all bacteria contain both DNA DSB repair pathways, including *E. coli*, which lacks the NHEJ genes *ku* and *ligD* [37].

Studies have previously shown that DNA damage repair systems and the SOS response are important for *E. coli* persister survival during exponential- and stationary-phase FQ treatment [38–43]. For example, Dörr and colleagues found that loss of *recA* or *recB* or inhibition of SOS induction reduced persister levels compared to wild-type (WT) after CIP treatment of exponentially-growing cells [38]. Additional studies have shown that nongrowing *E. coli* mutants deficient in HR functionalities or unable to induce the SOS response have significantly lower persistence than WT [39,40,43,44]. Importantly, it has been demonstrated that FQ persisters experience DNA damage and that damage repair via HR occurs during recovery from treatment [15,40–42,45]. Collectively, these studies have shown that DNA damage repair is a key factor contributing to persister survival in *E. coli* across a variety of growth and nutrient conditions.

However, the importance of DNA damage repair for FQ persistence has been sparingly studied in *P. aeruginosa* [46], a clinically relevant pathogen that has notable differences in its DNA damage repair and response machinery compared to that of *E. coli* [24–27,37,47]. In *P. aeruginosa*, there are 15 genes in the SOS regulon [27], which is in contrast to more than 60 genes in that of *E. coli* [24–26,28,47]. Furthermore, there are multiple DNA damage repair genes, such as *uvrA, uvrB, uvrD, ruvA,* and *ruvB,* that *P. aeruginosa* contains but are not SOS-regulated like they are in *E. coli* [24–27]*.* Further, *P. aeruginosa* contains both HR and NHEJ machinery, whereas *E. coli* does not contain NHEJ genes [32,35,37,48]. Given these differences, we hypothesized that the role of DNA damage repair in *E. coli* FQ persister survival may differ from that of *P. aeruginosa* FQ persisters.

Here we sought to examine the importance of DNA damage repair and the SOS response to *P. aeruginosa* FQ persistence and compare it to that of *E. coli* under the same conditions. We found that *P. aeruginosa* exhibited persistence to CIP during both stationary- and exponential-phase treatment and confirmed that regrowth of persisters produced a population with the same survival characteristics as the original cultures. Using deletion mutants deficient in HR (Δ*recA* and Δ*recB*), we found that HR is important for persistence of stationary-phase *P. aeruginosa* to CIP but dispensable to CIP persisters in exponential-phase cultures. In contrast, HR was important for the survival of stationary- and exponential-phase *E. coli* during CIP persistence assays. The importance of the SOS response mirrored that of HR (*P. aeruginosa* – stationary-phase; *E. coli* – stationary- and exponential-phases), whereas we found that NHEJ was not important to CIP persisters of *P. aeruginosa*. Interestingly, Ku and LigD overexpression was toxic to *P. aeruginosa*, which was not anticipated*.* To assess the generality of these findings to other FQs, we used LEV and observed that HR and the SOS response were important to LEV persistence of stationary-phase *P. aeruginosa* cultures, but not exponential-phase populations. In addition, we assessed the translatability of observations to clinical isolates with the use of *P. aeruginosa* strain MRSN 1612 [49], and we found that *recA* was important to CIP persisters in stationary-phase cultures yet dispensable to those in exponential-phase populations. Overall, these results show that HR and the SOS response are important for the survival of *P. aeruginosa* persisters treated with FQ during stationary-phase but not during exponential-phase, and that the importances of these systems are only partially conserved between *P. aeruginosa* and the model organism *E. coli*. This conservation between *P. aeruginosa* and *E. coli* persister survival mechanisms during stationary-phase treatment suggests that similar strategies for persister eradication may be possible across the two different species. However, distinct results in exponential-phase suggest that caution should be used when extrapolating understanding of persistence in one species to another.

## Results

### HR is important for FQ persistence of stationary-phase *P. aeruginosa* populations

**Fig 1:**
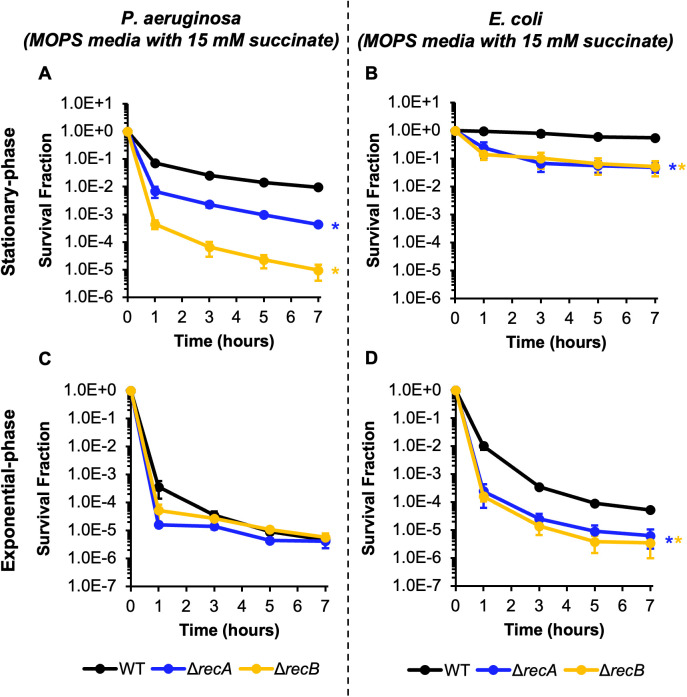
Contribution of HR to CIP persistence in stationary- and exponential-phase populations of *P. aeruginosa* and *E. coli.* **(A and C)** *P. aeruginosa* PAO1 WT and HR mutants were grown to **(A)** stationary phase (24 h) or **(C)** an OD_600_ ~ 0.2 in MOPS minimal media with succinate and then treated with 10 μg/mL CIP. **(B and D)** *E. coli* MG1655 WT and HR mutants were grown to **(B)** stationary phase (28 h) or **(D)** an OD_600_ ~ 0.2 in MOPS minimal media with succinate and then treated with 10 μg/mL CIP. Samples were taken at the indicated time points, washed, and plated on LB agar to quantify survivors. Data points reflect the means of at least 3 biological replicates. Error bars indicate standard errors of the means. One-way ANOVA with post-hoc Tukey tests were performed on t = 7 h log-transformed survival fractions to assess significance. *Asterisks denote statistical significance (defined as p ≤ 0.05) with respect to WT.

WT *P. aeruginosa* PAO1 were grown in minimal media with 15 mM succinate as the sole carbon source for 24 hours and then treated with 10 μg/mL CIP for 7 hours (MICs for all strains are shown in S1 Table). This resulted in biphasic killing with a survival of ~1% after 7 hours of treatment (Fig 1A). When we harvested the colonies that survived 7 hours of CIP treatment, regrew them, and subjected them to a second round of CIP treatment, the persister-derived population exhibited the same killing dynamics as the parent population (S1A Fig). Cultures treated with the solvent confirmed that the observed decreases in survival were due to CIP treatment (S1B Fig). These results demonstrate that in the conditions used in this study, *P. aeruginosa* strain PAO1 exhibits persistence to CIP.

To determine if HR is important for persisters to survive FQ treatment, we examined the survival of HR-deficient mutants, Δ*recA* and Δ*recB*, treated with 10 μg/mL CIP. When *P. aeruginosa* Δ*recA* and Δ*recB* mutants were grown for 24 hours in minimal media with 15 mM succinate and then treated with CIP, the persister levels of the Δ*recA* and Δ*recB* mutants decreased significantly by ~20- and ~1,000-fold compared to WT, respectively (Fig 1A). Chromosomal complementation of these genes restored persistence to WT or near-WT levels (S2A, S2B Fig), and solvent-treated controls exhibited minimal changes in culturability (S2C, S2D, S3A Figs). Notably, we observed that *P. aeruginosa* Δ*recA* and Δ*recB* mutants grew more slowly than WT and enter stationary-phase 1 and 5 hours later than WT, respectively (S4A, S4B Fig). To confirm that the reduced persister levels of these mutants were not due to reduced amounts of time in stationary-phase, we increased the period of incubation prior to CIP treatment to match the duration of time spent in stationary-phase prior to treatment. When we treated Δ*recA* and Δ*recB* mutants with CIP after increasing the period of incubation by 1 and 5 hours, respectively, *P. aeruginosa* Δ*recA* persister levels did not change (S5A Fig), whereas persister levels of *P. aeruginosa* Δ*recB* increased ~10-fold such that they approached those of Δ*recA* (S5B Fig). Solvent-treated controls exhibited minimal changes in survival fractions (S5C, S5D Fig). In addition, we performed CIP persistence assays at the same fold-MIC concentrations for all strains (40xMIC: 10 μg/mL for WT, 2 μg/mL for Δ*recA*, 1.25 μg/mL for Δ*recB*) and observed significant decreases in persistence for Δ*recA* and Δ*recB* compared to WT of ~50-fold and ~200-fold, respectively, which were comparable in magnitude to those observed at the same absolute concentration of 10 μg/mL CIP (S6A, S6B Fig). These results indicate that HR is important for *P. aeruginosa* persisters to survive stationary-phase treatment with CIP.

To directly compare FQ persistence of *P. aeruginosa* to that of *E. coli*, we grew and treated *E. coli* in the same minimal succinate media. Succinate is the preferred carbon source for *P. aeruginosa*, and *E. coli* can grow with succinate as the sole carbon source, but most work on FQ persistence with *E. coli* in minimal media has been performed with glucose as the sole carbon source [15,40,41,43,45,50]. We treated *E. coli* cultures with CIP after 28 hours of growth in 15 mM succinate so that WT *E. coli* cultures were in stationary-phase for the same amount of time as WT *P. aeruginosa* cultures prior to treatment (S4A–S4D Fig). WT *E. coli* exhibited almost complete tolerance when treated with 10 μg/mL CIP, with the majority of the population surviving after 7 hours of treatment (Fig 1B). This is consistent with previous work demonstrating the low efficacy of CIP against stationary-phase *E. coli* [51]. Indeed, when WT *E. coli* cultures were grown in minimal media with 10 mM glucose, its typical sole carbon source, to stationary-phase and treated with CIP, similarly high survival was observed (S4E, S4F, S7A Figs). Solvent-treated controls exhibited minimal changes in survival fraction (S3B, S7B Figs). These results indicate that in the same culture conditions, *P. aeruginosa* produces fewer persisters than *E. coli*. When *E. coli* Δ*recA* and Δ*recB* mutants were assayed, survival decreased significantly by ~10-fold compared to WT (Fig 1B). *E. coli* Δ*recA* and Δ*recB* grew more slowly than WT, entering stationary-phase 1 and 2 hours later than WT, respectively (S4C, S4D Fig). When we extended the incubation prior to treatment of these strains to match the period of time in stationary-phase to that of WT prior to treatment, Δ*recA* persister levels did not change, and Δ*recB* persister levels decreased ~7-fold, confirming that the reduced survival compared to WT was not due to less time in stationary-phase prior to treatment (S5E, S5F Fig). Controls treated with solvent confirmed that the observed killing was due to CIP (S3B, S5G, S5H Figs). These results demonstrate the importance of HR to the survival of stationary-phase *E. coli* during CIP persistence assays, which is consistent with previous studies performed with different media and FQ treatment conditions [15,40–42].

### HR does not contribute to FQ persistence of growing *P. aeruginosa* populations

Next, we sought to evaluate the importance of HR for FQ persistence of exponentially-growing *P. aeruginosa* and compare it to that of *E. coli*. We treated *P. aeruginosa* and *E. coli* strains grown to exponential-phase in minimal media containing 15 mM succinate with 10 μg/mL CIP for 7 hours. For *P. aeruginosa*, persister levels of Δ*recA* and Δ*recB* were similar to those of WT after 7 hours of treatment, which suggested that HR is not important for FQ persistence of exponentially-growing *P. aeruginosa* (Fig 1C). Conversely, *E. coli* Δ*recA* and Δ*recB* exhibited ~10-fold reductions in survival compared to WT, which suggested that HR contributed to FQ persistence within those growth conditions (Fig 1D). These results are consistent with previous studies of *E. coli* FQ persistence in exponential-phase cultures where different treatment conditions were used [38,39]. It is notable that despite equivalent persister levels at 7 hours of treatment for *P. aeruginosa* WT and its HR mutants, the initial death rates of exponential-phase cultures of Δ*recA* and Δ*recB* treated with CIP were more precipitous than those of WT (Fig 1C). This reflected a difference in susceptibility to CIP of normal cells in those populations compared to those of WT since the difference occurred in the first phase of killing [2]. However, after sufficient time had elapsed to be well within the second phase of killing (7 hours), which is where colony forming unit (CFU) measurements reflect persister abundances [2], the survival fractions were equivalent (Fig 1C), and thus the susceptibility of normal cells in those populations did not correlate with the persister levels. When CIP persisters from exponentially-growing *P. aeruginosa* were regrown and assayed again, persister levels were equivalent to those of the original culture (S1C Fig), and solvent-treated cultures did not exhibit decreases in survival (S1D, S3C, S3D Figs). These results suggest that HR machinery is not uniformly important for FQ persistence in growing cultures because loss of HR in *P. aeruginosa* did not affect persister levels, whereas HR machinery was important for *E. coli* persistence in identical culturing conditions.

### *P. aeruginosa* SOS response contributes to FQ persistence only during stationary-phase

**Fig 2:**
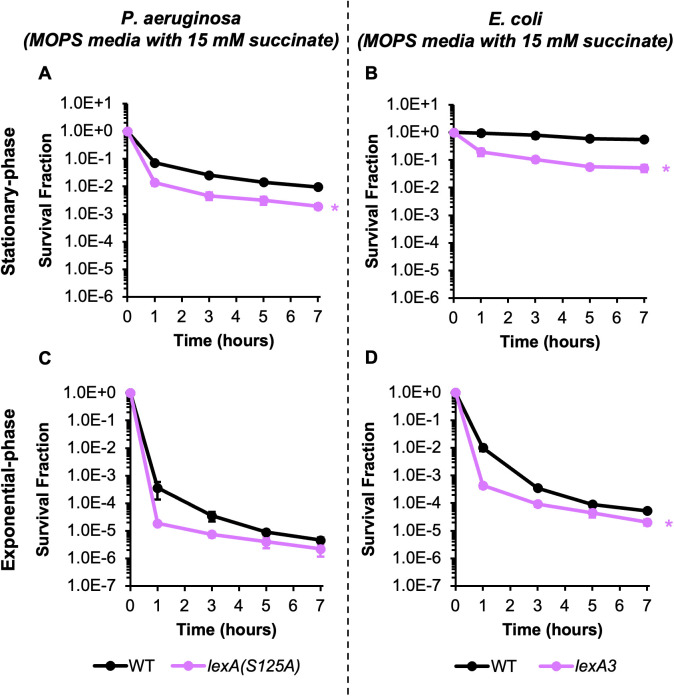
Importance of SOS induction for CIP persister survival mirrors that of HR in *P. aeruginosa* and *E. coli.* **(A and C)** *P. aeruginosa* PAO1 WT and its SOS mutant were grown to **(A)** stationary phase (24 h) or **(C)** an OD_600_ ~ 0.2 in MOPS minimal media with succinate and then treated with 10 μg/mL CIP. **(B and D)** *E. coli* MG1655 WT and its SOS mutant were grown to **(B)** stationary phase (28 h) or **(D)** an OD_600_ ~ 0.2 in MOPS minimal media with succinate and then treated with 10 μg/mL CIP. Samples were taken at the indicated time points, washed, and plated on LB agar to quantify survivors. Data points reflect the means of at least 3 biological replicates. Error bars indicate standard errors of the means. Two-tailed t-tests with unequal variances were performed on t = 7 h log-transformed survival fractions to assess significance. *Asterisks denote statistical significance (defined as p ≤ 0.05) with respect to WT.

The SOS regulon of *P. aeruginosa* consists of only 15 genes, including those encoding DNA damage repair functions like *recA* and *imuABC* [27,52], whereas the SOS regulon of *E. coli* is up to four times larger (as many as 60 genes have been demonstrated to be LexA-regulated) and includes a larger swath of DNA damage repair machinery, such as *uvrA, uvrB, uvrD,* and *ruvAB* [24–26,28,47]. We sought to evaluate the importance of the SOS response for CIP persistence of *P. aeruginosa* and compare it to that of *E. coli*. To accomplish that, we generated an uncleavable LexA mutant in *P. aeruginosa*, *lexA(S125A)*, and confirmed with flow cytometry that its SOS response was highly attenuated (S8 Fig). Stationary-phase *P. aeruginosa lexA(S125A)* exhibited a significant 5-fold decrease in persister levels compared to WT when treated with 10 μg/mL CIP for 7 hours (Fig 2A), and when assays were performed at the same fold-MIC concentrations for both strains (40xMIC: 10 μg/mL for WT, 2.5 μg/mL for *lexA(S125A)*), a significant reduction in persister levels of ~30-fold for *lexA(S125A)* was observed in comparison to WT (S6C Fig). For *E. coli*, we assayed *lexA3*, which is an uncleavable LexA mutant in *E. coli* that cannot activate the SOS response [40,53,54], in comparison to WT and observed a significant 10-fold decrease in survival after 7 hours of CIP treatment in stationary-phase (Fig 2B). We noticed that *E. coli lexA3* enters stationary-phase later than WT (S4C, S4D Fig). To account for that, *E. coli lexA3* was treated with CIP after an equivalent period of time in stationary-phase as WT, and the persister levels of *lexA3* did not change compared to the standard incubation, which confirmed that the reduced survival was not due to different periods of time in stationary-phase (S5I Fig). Solvent-treated controls confirmed that these decreases in persister levels were due to CIP treatment (S3A, S3B, S5J Figs). When exponentially-growing cultures were treated with CIP for 7 hours, *P. aeruginosa lexA(S125A)* persister levels were not significantly different from those of WT, whereas *E. coli lexA3* exhibited a significant ~2.5-fold reduction in persister levels compared to WT in the minimal succinate media used here (Fig 2C, 2D). The initial death rates for *P. aeruginosa* WT and its *lexA* mutant were different here, but much like the HR mutants, that difference in normal cell susceptibility was not reflected in the persister levels during the second phase of CIP killing (Fig 2C). Solvent-treated cultures did not exhibit decreases in survival (S3C, S3D Fig). Collectively, these results indicate that when *P. aeruginosa* and *E. coli* are grown with succinate as the sole carbon source, inhibition of SOS activation results in similarly decreased survival during stationary-phase FQ treatment. However, different dependencies are observed during exponential-phase treatment, where the SOS response is not important for the survival of *P. aeruginosa* persisters in exponential-phase cultures but is moderately important for *E. coli* persistence.

### Loss of NHEJ does not impact *P. aeruginosa* persister levels

**Fig 3:**
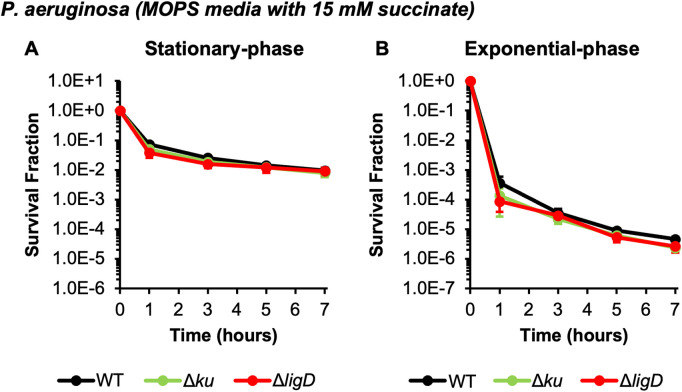
Loss of NHEJ machinery does not alter *P. aeruginosa* persister levels. *P. aeruginosa* PAO1 WT and NHEJ mutants were grown to **(A)** stationary phase (24 h) or **(B)** an OD_600_ ~ 0.2 in MOPS minimal media with succinate and then treated with 10 μg/mL CIP. Samples were taken at the indicated time points, washed, and plated on LB agar to quantify survivors. Data points reflect the means of at least 3 biological replicates. Error bars indicate standard errors of the means. One-way ANOVA with post-hoc Tukey tests were performed on t = 7 h log-transformed survival fractions to assess significance. No statistical significance was found.

*P. aeruginosa*, unlike *E. coli*, contains the machinery to perform NHEJ: namely, the proteins Ku (PA2150) and LigD (PA2138) [35,37]. We assessed the importance of NHEJ to FQ persistence of *P. aeruginosa* during both stationary- and exponential-phase using deletion mutants. As shown in Fig 3, Δ*ku* and Δ*ligD* did not exhibit reduced CIP persister levels compared to WT during stationary- or exponential-phase, and treatment with solvent showed minimal changes in culturability (S3A, S3C Fig). Further, even in the absence of HR, loss of NHEJ (Δ*recA* Δ*ku*) did not alter killing by CIP in either growth phase compared to loss of HR alone (S9 Fig). These results indicate that NHEJ is dispensable for FQ persistence of *P. aeruginosa* in both stationary-phase and exponential-phase populations.

### Overexpressing NHEJ machinery is toxic to *P. aeruginosa*

**Fig 4:**
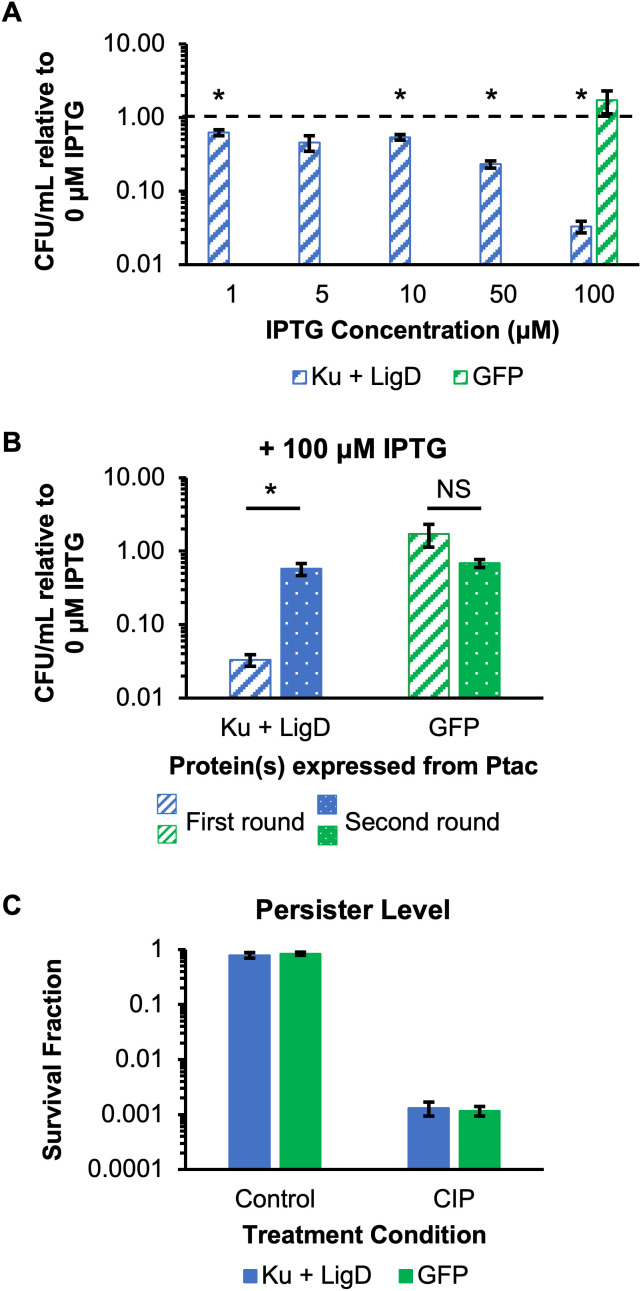
Overexpression of NHEJ machinery is toxic to *P. aeruginosa* but does not impact FQ persister levels under non-toxic induction. **(A)** *P. aeruginosa* PAO1 overexpression strains were inoculated into MOPS minimal media with succinate and 30 μg/mL gentamicin (for plasmid retention) from overnight growth in LB to OD_600_ ~ 0.01. After 4 hours of growth, IPTG was added at the indicated concentrations. After 20 additional hours of incubation, samples were washed and plated on LB agar with 30 μg/mL gentamicin and IPTG at the same concentration as liquid cultures. CFUs were quantified after 36 hours of incubation. **(B)** Survivors from the first round of 100 μM IPTG induction were harvested, stocked, and used in a second round of IPTG induction that was identical to the first. **(C)** The impact of overexpression on *P. aeruginosa* persister levels was examined using a non-toxic induction protocol (Method II in S11B Fig). *P. aeruginosa* PAO1 containing pGL11 or pGL12 were grown to stationary phase (24 h) in MOPS minimal media with succinate and 30 μg/mL gentamicin followed by treatment with solvent (control) or 10 μg/mL CIP. At t = 0 h and t = 7 h, samples were taken, washed, and plated on filters on LB agar with 100 μM IPTG and 30 μg/mL gentamicin. After 2 hours of incubation, filters were transferred to plates without IPTG and incubated for an additional 18-22 hours. Data reflect the means of at least 3 biological replicates. Error bars indicate standard errors of the means. **(A and B)** *Asterisks denote statistical significance of the log-transformed relative culturability (induction/no induction) compared to a value of log(1) using two-tailed t-tests with unequal variances and a Bonferroni correction **(A)**, or between first and second round log-transformed relative culturability levels using two-tailed t-tests with unequal variances **(B)**. NS denotes no statistical significance detected.

Based on the above results, we hypothesized that low native expression of Ku and LigD might be limiting their ability to enable persister survival. To test this hypothesis, we constructed a synthetic operon to express Ku and LigD from the IPTG-inducible P_*tac*_ promoter in WT *P. aeruginosa* and confirmed that induction with IPTG resulted in higher levels of Ku and LigD compared to an empty vector control and a strain expressing superfolder (sf)GFP using a protein gel and mass spectrometry (S10 Fig). We first investigated overexpression of Ku and LigD in WT *P. aeruginosa* in untreated conditions. When Ku and LigD expression was induced for 20 hours in liquid cultures and then 36 hours on LB agar plates with IPTG (S11A Fig), culturability decreased significantly with IPTG induction at concentrations as low as 1 μM IPTG compared to no induction (Fig 4A). Reduced culturability was not observed when the strain expressing GFP was induced with 100 μM IPTG (Fig 4A). These results suggested that Ku and LigD overexpression was toxic to *P. aeruginosa.*

Given the potential toxicity, we wondered if culturable cells in Ku and LigD overexpression samples may have obtained mutations that reduced Ku and/or LigD expression/activity. To assess that, we harvested colonies that survived one round of 100 μM IPTG induction, stocked them, and performed a second round of induction with 100 μM IPTG identical to the first round. As shown in Fig 4B, the culturability after a second round of induction increased >10-fold in the strain expressing Ku and LigD, but not in the strain expressing GFP. These results indicated that after a single round of IPTG induction, culturable cells that carried the Ku and LigD overexpression vector had acquired a heritable change that allowed them to survive induction. To address this technical hurdle, we evaluated different induction conditions for their impact on culturability and identified one that resulted in no significant difference in culturability relative to that of the no induction control: induction with 100 μM IPTG only during the first two hours on LB agar plates (S11B, S11C Fig, see S1 Methods), which would correspond to the recovery period of FQ persisters [40,41]. Induction with 100 μM IPTG was shown to result in expression of Ku and LigD (Fig 4A), and induction during the first two hours of cultivation on LB agar would ensure that Ku and LigD are expressed during the time period when DNA repair would be needed following FQ treatment [15,40,41]. Using this induction method, we assessed the impact of Ku and LigD overexpression on FQ persistence. As shown in Fig 4C, overexpression of Ku and LigD resulted in the same survival as overexpression of GFP after 7 hours of CIP treatment. These results demonstrated that overexpressing Ku and LigD such that no toxic effects were observed does not impact survival of stationary-phase *P. aeruginosa* treated with CIP, and together with the deletion mutant data from above suggested that Ku and LigD are not repairing DNA damage sustained from FQ treatment.

### Impacts of HR and SOS on *P. aeruginosa* persistence to another FQ and in a clinical isolate

**Fig 5:**
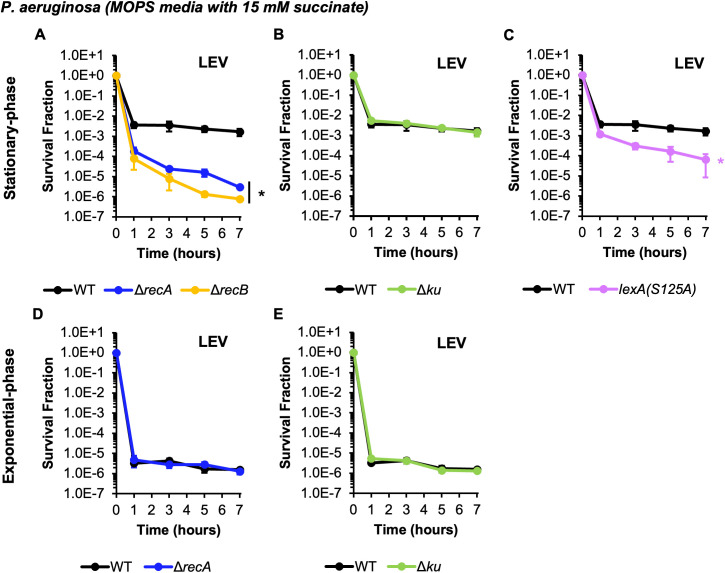
Contribution of DNA damage repair to *P. aeruginosa* persister survival extends to LEV. *P. aeruginosa* PAO1 WT and DNA damage repair mutants were grown to either **(A-C)** stationary-phase (24 h) or **(D and E)** an OD_600_ ~ 0.2 in MOPS minimal media with succinate and then treated with 40 μg/mL LEV. Samples were taken at the indicated time points, washed, and plated on LB agar to quantify survivors. Solvent-treated controls are shown in S12 Fig. Data points reflect the means of **(A-C)** 3 biological replicates or **(D and E)** at least 2 biological replicates. Error bars indicate standard errors of the means. **(A)** One-way ANOVA with post-hoc Tukey tests were performed on t = 7 h log-transformed survival fractions to assess significance. **(B and C)** Two-tailed t-tests with unequal variances were performed on t = 7 h log-transformed survival fractions to assess significance. *Asterisks denote statistical significance (defined as p ≤ 0.05) with respect to WT.

**Fig 6:**
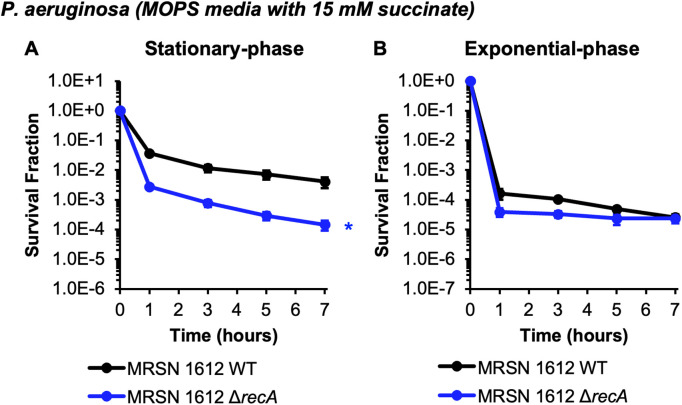
HR is important for CIP persistence of *P. aeruginosa* clinical isolate MRSN 1612 only during stationary-phase. *P. aeruginosa* MRSN 1612 WT and Δ*recA* were grown to either **(A)** stationary-phase (24 h) or **(B)** an OD_600_ ~ 0.2 in MOPS minimal media with succinate and then treated with 10 μg/mL CIP. Samples were taken at the indicated time points, washed, and plated on LB agar to quantify survivors. Data points reflect the means of **(A)** at least 3 biological replicates or **(B)** at least 2 biological replicates. Error bars indicate standard errors of the means. **(A)** A two-tailed t-test with unequal variances was performed on t = 7 h log-transformed survival fractions to assess significance. *Asterisk denotes statistical significance (defined as p ≤ 0.05) with respect to WT.

Since the above experiments were performed with one FQ, CIP, and one strain of *P. aeruginosa*, PAO1, we sought to assess the generality of our observations with a different FQ and different *P. aeruginosa* strain. We performed experiments with LEV and observed dependencies of persistence on DNA repair genes that were analogous to those we observed with CIP (Fig 5). Specifically, significant reductions in persister levels were observed for stationary-phase populations of Δ*recA*, Δ*recB*, and *lexA(S125A)* in comparison to WT, whereas those for Δ*ku* were comparable to WT (Fig 5A–5C); and persister levels in exponential-phase cultures of Δ*recA* and Δ*ku* were found to be similar to those of WT (Fig 5D, 5E). To assess generality to other *P. aeruginosa* strains, we selected *P. aeruginosa* MRSN 1612, which is a clinical isolate that was collected as part of a global surveillance program [49]. Notably, PAO1 is a member of clade A (*exoS*+ , *exoU*-) and MRSN 1612 is a member of clade B (*exoS*-, *exoU*+), which represent the two largest *P. aeruginosa* clades associated with infections in humans [49]. We constructed a Δ*recA* mutant of MRSN 1612 using allelic exchange [55] and then performed CIP persistence assays on it and its WT parent. Fig 6 depicts a significant ~30-fold reduction in CIP persistence in stationary-phase cultures of Δ*recA* compared to WT, whereas loss of *recA* had a negligible impact on CIP persistence in exponential-phase. Collectively, these results demonstrate that observations made with CIP were consistent with those using LEV, another FQ, and that the selective importance of *recA* to CIP persistence in stationary-phase, which we initially observed with *P. aeruginosa* PAO1, was also present in a recent, unrelated clinical isolate.

## Discussion

*P. aeruginosa* is a common cause of hospital-acquired infections that is also often associated with lung infections in cystic fibrosis patients [10–12]. CIP, an FQ that is approved to treat *P. aeruginosa* infections [18], is considered a critically important antimicrobial by the World Health Organization (WHO), which has given it the “watch group” classification due to its importance and propensity for resistance development [56,57]. Importantly, studies in *E. coli* have shown that FQ persistence can enhance resistance development [15,17,58,59]. During FQ treatment, persisters can experience DNA damage that is repaired by HR and leads to activation of the SOS response, resulting in increased abundances of error-prone DNA polymerases that can introduce mutations that confer resistance [15,40]. Additionally, recent work in *E. coli* has shown that Mfd, a protein involved in transcription-coupled repair, contributes to resistance development from FQ persisters, further demonstrating the role of DNA damage repair in resistance arising from persisters [59]. Therefore, understanding how persisters survive antibiotic treatment can eliminate persistence as a cause of treatment failure and slow the development of antibiotic resistance as a result. The contributions of DNA damage repair and SOS induction to persister survival in bacteria other than the model organism *E. coli* have remained ill-defined. This knowledge gap motivated us to investigate the roles of DNA damage repair and SOS induction in *P. aeruginosa* persisters to FQs.

To assess whether DNA repair is used by *P. aeruginosa* persisters to survive FQs*,* we performed persistence assays on *P. aeruginosa* strains deficient in HR, SOS induction, and NHEJ. We also compared those results to analogous mutants of *E. coli* grown and treated in the same conditions to assess whether trends were conserved between the two Gram-negative bacterial species (see Table 1 for a summary of results). First, we observed that while *P. aeruginosa* exhibited biphasic killing during stationary-phase treatment with 10 μg/mL CIP (~1% survival after 7 hours), *E. coli* exhibited near complete tolerance under the same treatment conditions (Fig 1A, 1B). This finding is notable when considering that the MIC of *P. aeruginosa* is > 10-fold higher than that of *E. coli* (S1 Table), which illustrates that metrics of resistance (MIC) do not always correlate with levels of tolerance. When loss of HR machinery was assayed, Δ*recA* and Δ*recB* produced fewer CIP persisters in *P. aeruginosa* and *E. coli* compared to their respective WT controls in stationary-phase (Fig 1A, 1B), which was a phenomenon that was also observed with LEV and Δ*recA* and Δ*recB* of *P. aeruginosa* (Fig 5A). When SOS induction was quenched with uncleavable LexA mutants, reduced CIP persister levels were also found for *P. aeruginosa* and *E. coli* in stationary-phase (Fig 2A, 2B), which was a result mirrored with LEV treatment of *P. aeruginosa* (Fig 5C). These results, which are consistent with previous work in *E. coli* [40,42], indicate that HR and the SOS response are both used by stationary-phase FQ persisters in these different Gram-negative species. On the other hand, the importance of these DNA damage repair systems for CIP persistence was not conserved in exponential-phase, where loss of HR, and to a lesser extent SOS induction, reduced persister levels for *E. coli* but not *P. aeruginosa* (Figs 1C, 1D, 2C, 2D). Analogously, Δ*recA* and WT persister levels to LEV were equivalent in exponential-phase *P. aeruginosa* populations (Fig 5D). The results for *E. coli* agree with previous work in different conditions [38], whereas those for *P. aeruginosa* reveal that HR and SOS induction do not contribute to persister levels in exponentially-growing cultures. Two scenarios that could underlie the lack of involvement of HR and SOS induction in FQ persistence of exponential-phase *P. aeruginosa* are that persisters experience DNA damage that is repaired by other systems or FQs fail to corrupt their targets and cause DNA damage. Indeed, components of other DNA damage repair systems, such as those of nucleotide excision repair, have been shown to contribute to FQ persistence in *E. coli* [39,43,44,59]. Alternatively, lack of target corruption could occur through DNA gyrase and topoisomerase IV inactivity, which could be caused by low ATP levels [20,60], or active efflux, which could limit antibiotic-target interactions [61]. For example, Cameron and coworkers demonstrated that reducing ATP levels with arsenate increased FQ persister levels of exponential-phase *P. aeruginosa* [62]. Further, Pu and coworkers showed that efflux plays a role in persistence of *E. coli* populations [63]. Given the extremely low frequency of FQ persisters in growing *P. aeruginosa* cultures (<1 in 100,000, Figs 1 and 5), methods to observe low ATP levels or efflux in persisters with time-lapse fluorescence microscopy or fluorescence-activated cell sorting (FACS) would be extremely difficult. However, with the advent of genomic recorders that function in persisters [64], we postulate that it will not be long until a variety of physiological traits (*e.g.,* SOS induction, increased expression of efflux pumps, low energy levels) can be recorded into the DNA of persisters before, during, or after treatment, which can subsequently be read from the DNA of their progeny after regrowth.

**Table 1:** Growth phases in which indicated genes impact CIP persistence.

Gene name	*P. aeruginosa*	*E. coli*
*recA*	Stationary	Stationary and Exponential
*recB*	Stationary	Stationary and Exponential
*lexA*	Stationary	Stationary and Exponential
*ku*	None	N/A
*ligD*	None	N/A

N/A: not applicable

Our results showing reduced stationary-phase persistence of *P. aeruginosa* Δ*recA* to CIP and LEV in comparison to WT differ from recent work with LEV in different growth and treatment conditions [46]. Specifically, the media, length of cultivation, concentration of FQ, and length of treatment all differed, which likely explains the variable dependencies on *recA* [46]. However, it is worth noting that the previous work illustrating insignificant survival differences between WT and Δ*recA* when LEV was used for treatment agree with our results with CIP and LEV in exponential-phase cultures (Figs 1C and 5D) and reinforce the notion that *recA* is not universally important for persistence to FQs [46].

Persister studies have often focused on *E. coli,* so the role of NHEJ, for which *E. coli* does not have the machinery [37], has remained ill-defined. We examined the importance of NHEJ to FQ persistence by knocking out *P. aeruginosa* NHEJ genes *ku*, which has been shown *in vitro* to bind broken DNA ends and protect them from degradation [65], or *ligD*, which has been shown in *in vitro* studies to have nuclease, polymerase, and ligase activity [35,36,66]. Loss of NHEJ did not alter persister levels during CIP or LEV treatment in either exponential- or stationary-phase, even in the absence of HR (Δ*recA* Δ*ku*) (Figs 3, 5B, 5E; S9 Fig). Those results raised the question of whether NHEJ had the potential to impact persistence, which we investigated with an overexpression construct. We observed that overexpression of Ku and LigD had a toxic effect on *P. aeruginosa* (Fig 4A). Although the mechanism of this toxicity is beyond the scope of this study, we suspect that Ku and LigD can act on spontaneous DNA DSBs [67,68] to prevent their repair by HR and produce lethal mutagenic lesions. In support of this, it has been shown that the protein Gam from phage Mu, which is an ortholog of Ku that also binds to double-stranded ends of DNA, can inhibit RecBCD activity in *E. coli* [67,69,70]. Furthermore, *in vitro* work has demonstrated that *P. aeruginosa* LigD activity can be mutagenic [36], and previous work has shown that chromosomal deletions in *P. aeruginosa* can arise due to NHEJ repair of DSBs generated by MutL [71], a protein involved in mismatch repair [72,73]. Additional work is needed to understand why overexpressing Ku and LigD is toxic under normal growth conditions and uncover the role of NHEJ in DNA DSB repair in *P. aeruginosa*.

In conclusion, we have shown that in *P. aeruginosa*, the roles of different DNA repair systems in FQ persister survival depend on the growth phase during treatment. Specifically, HR and the SOS response are important for stationary-phase *P. aeruginosa* populations to survive treatment, which is similar to *E. coli*. However, unlike *E. coli*, neither of these systems contributes to persister survival during FQ treatment of exponentially-growing populations, and NHEJ does not play a role in persister survival regardless of the growth phase during FQ treatment. The conserved roles of DNA repair for stationary-phase FQ persistence suggest that HR and the SOS response could serve as therapeutic targets to reduce persistence and incidences of FQ treatment failure in diverse Gram-negative bacteria. This work also signals the importance of extending persister studies beyond *E. coli* because the relevance of mechanisms between species is not always comparable.

## Materials and methods

### Chemicals and growth media

All strains and plasmids used in this study are listed in S2 Table. All chemicals and growth media were purchased from Sigma Aldrich or Fisher Scientific, unless otherwise specified. All media were prepared using distilled water purified with a MilliQ system to a resistivity of 18.2 MΩ. 3-(N-morpholino)propanesulfonic acid (MOPS) minimal media supplemented with 15 mM succinate was prepared by mixing 10xMOPS buffer (MOPS Minimal Media Kit M2106, Teknova), 0.132 M K_2_HPO_4_ (MOPS Minimal Media Kit M2106, Teknova), and 1 M sodium succinate (sterilized by filtration with a 0.22 μm syringe filter) with autoclaved MilliQ water to achieve a composition of 1xMOPS buffer, 0.00132 M K_2_HPO_4_, and 15 mM succinate. MOPS media was sterilized by vacuum filtration through a 0.22 μm filter. Phosphate buffered saline (PBS) was prepared by mixing sterile 10xPBS (prepared by mixing powdered PBS (Fisher Scientific, BP665-1) with MilliQ water according to the manufacturer’s instructions and autoclaving) with autoclaved MilliQ water to achieve a 1xPBS solution. 1xPBS was sterilized by vacuum filtration through a 0.22 μm filter. Luria Bertani (LB) media was prepared by mixing 10 g/L tryptone, 5 g/L yeast extract, and 10 g/L NaCl with MilliQ water and autoclaving to achieve sterilization, and LB agar was prepared in the same way with agar added at a concentration of 15 g/L. Three hundred mM sucrose was prepared by dissolving sucrose in autoclaved MilliQ water and filtering with a 0.22 μm syringe filter. Vogel-Bronner minimal medium (VBMM) agar, which was used for generation of mutants via the two-step allelic exchange method [55], was prepared by diluting 10xVBMM salts (2 g/L MgSO_4_·7H_2_O, 20 g/L citric acid, 100 g/L K_2_HPO_4_, and 35 g/L NaNH_4_HPO_4_·4H_2_O in MilliQ water titrated to pH ~ 7.0 and sterilized by vacuum filtration) in autoclaved agar (15 g agar/0.9 L water) to achieve 1xVBMM in 15 g/L agar. To prepare no salt LB (NSLB) + 15% (w/v) sucrose agar, first tryptone, yeast extract, and agar were mixed with MilliQ water to achieve 10 g/0.7 L, 5 g/0.7 L, and 15 g/0.7 L of each component, respectively, and that mixture was autoclaved. Then, 50% (w/v) sucrose solution (sterilized by filtration through 0.22 μm filter) was added once the agar had cooled but remained liquid to achieve 15% sucrose in 10 g/L tryptone + 5 g/L yeast extract + 15 g/L agar. NSLB + 300 mM sucrose agar was prepared similarly, except NSLB agar was first prepared as 10 g/0.8 L tryptone, 5 g/0.8 L yeast extract, and 15 g/0.8 L agar in MilliQ water and autoclaved, and then 1.5 M sucrose (sterilized by filtration with 0.22 μm filter) was added to achieve 300 mM sucrose in NSLB + 15 g/L agar. For plasmid retention and mutant selection with *P. aeruginosa*, antibiotics were used at the following concentrations: 30 μg/mL gentamicin and 200–300 μg/mL carbenicillin where specified. For plasmid retention with *E. coli*, antibiotics were used at the following concentrations unless otherwise specified: 15 μg/mL gentamicin, 100 μg/mL ampicillin, and 50 μg/mL kanamycin. CIP for the persister assays was prepared by making a 5 mg/mL stock in 1 mL of 0.2 M HCl. This stock was then diluted 5-fold in MilliQ water to achieve a working stock of 1 mg/mL CIP. The solvent control was prepared in the same way but without CIP. LEV for the persister assays was prepared by making a 5 mg/mL stock in 1 mL of 20 mM NaOH, and the solvent control was prepared identically but without LEV. Isopropyl β-d-1-thiogalactopyranoside (IPTG) and arabinose were prepared as 1 M stocks in MilliQ water. Antibiotics, IPTG, and arabinose were sterilized by filtering with a 0.22 μm syringe filter.

### Mutant construction via λ Red recombination

To generate the PAO1 Δ*recA* and Δ*recB* mutants, a modified version of the λ Red recombination method described by Lesic and Rahme [74] was used. PAO1 WT or Δ*ku* was transformed with the plasmid pUCP18-RedS [74] via electroporation. To generate the deletion mutants in these strains, ~ 500–650 bp regions directly upstream and downstream of the *recA* or *recB* open reading frames were assembled with the gentamicin resistance cassette containing FRT sites via overlap PCR using the primers described in S3 Table. PCRs with extracted genomic DNA and plasmid DNA templates were performed with Phusion DNA polymerase (New England Biolabs, Inc.), and colony PCRs were performed with Taq DNA polymerase (New England Biolabs, Inc.). All overlap PCRs were performed using a 3-step PCR with Phusion DNA polymerase. Multiple simultaneous overlap PCR reactions were performed to ensure a high purified product concentration, all of which were run on an agarose gel and gel extracted (QIAquick Gel Extraction Kit, Qiagen) together. PAO1 WT (to generate PAO1 Δ*recA* and Δ*recB*) or Δ*ku* (to generate Δ*recA* Δ*ku*) containing pUCP18-RedS were grown from a single colony in 3 mL LB + 300 μg/mL carbenicillin at 37°C with shaking at 250 rpm. Once the growth was slightly turbid (~5–7 hours after inoculation), 10 mM arabinose was added to the culture to induce expression of the λ Red proteins, and the culture was grown at 37°C with shaking at 250 rpm for an additional ~16 hours. After ~16 hours, 3 mL cultures were made electrocompetent by washing 5–6 times in sterile 300 mM sucrose. After the last wash, the cell pellets were resuspended in ~80 μL of 300 mM sucrose, and >500 ng of the purified overlap PCR products were added to the cells. These mixtures were incubated at room temperature, electroporated, and then resuspended immediately in 1 mL LB. The cell suspensions were transferred to test tubes and incubated at 37°C with shaking at 250 rpm for ~10 minutes. To remove sucrose from cell suspensions (because the pUCP18-RedS plasmid expresses *sacB*), cells were washed twice in LB by pelleting cell suspensions at ~12,900 rpm (16,000 g) for 1 minute, removing all supernatant, and resuspending pellets in 1 mL LB. After two washes, cell suspensions were added to new test tubes and incubated at 37°C with shaking at 250 rpm for 3 hours. After 3 hours, the outgrowths were each concentrated to ~300 μL total volume and spread onto three LB + 30 μg/mL gentamicin agar plates. After 16–24 hours of incubation at 37°C, large colonies were selected, colony purified on LB + 30 μg/mL gentamicin agar plates, and checked for gene deletion via PCR with the primers listed in S3 Table. Deletions were confirmed with loss of internal PCR product and presence of a PCR product with a primer external to the gene being knocked out and a primer internal to the gentamicin resistance gene, *gmR*. Confirmed colonies were cured of pUCP18-RedS via sucrose counterselection by plating on NSLB + 300 mM sucrose, and then *gmR* was removed via Flp recombination. For Flp recombination, strains were transformed via electroporation with pGL04 and then plated on LB + 200 μg/mL carbenicillin + 1 mM IPTG agar plates to induce expression of Flp recombinase. After confirming loss of *gmR* via PCR and/or sequencing using the primer pairs listed in S3 Table (deletions were confirmed with loss of internal PCR product, loss of PCR product with gene external and *gmR* internal primers, and presence of external PCR product at the appropriate size) and confirming no growth with 30 μg/mL gentamicin, strains were streaked onto NSLB + 300 mM sucrose agar plates to cure of pGL04. Loss of pGL04 was confirmed by checking for inability to grow with 200 μg/mL carbenicillin.

### Mutant construction via two-step allelic exchange

*P. aeruginosa* PAO1 Δ*ku*, Δ*ligD*, and *lexA(S125A)* and MRSN 1612 Δ*recA* were generated using the two-step allelic exchange protocol described by Hmelo and colleagues [55]. Plasmids pGL05, pGL06, and pGL20 contain ~500–600 bp regions directly upstream and downstream of *ku*, *ligD*, and *recA*, respectively, assembled directly next to each other in pEXG2 [75], as described in S2 Table. Plasmid pGL08 (S2 Table) contains ~1,000 bp of *lexA* and surrounding DNA centered around the S125A mutation in *lexA* in pEXG2. Plasmids pGL05, pGL06, and pGL08 were transformed into *E. coli* strain S17-1 via electroporation for subsequent mating with P. aeruginosa PAO1. Plasmid pGL20 was transformed into S17-1 for subsequent mating with MRSN 1612. E. coli S17-1 expressing these donor plasmids and *P. aeruginosa* PAO1 WT or MRSN 1612 WT (for knocking out *recA*) were grown overnight from -80°C, 25% glycerol stocks in 2 mL LB (with 30 μg/mL gentamicin for the donor strains). After 16 hours, the donor strains were diluted 1:50 in 5 mL LB with 30 μg/mL gentamicin and grown at 37°C with shaking at 250 rpm to optical density measured at 600 nm (OD_600_) ~ 0.4-0.6. OD_600_ was measured with a Synergy H1 Hybrid Microplate Reader (Agilent Technologies). Simultaneously, the *P. aeruginosa* WT overnight cultures were diluted 1:1 in 1 mL LB and incubated at 42°C. Once the donor strains reached the target OD_600_, 1.5 mL of donor culture and 0.5 mL of *P. aeruginosa* WT culture were centrifuged at ~10,200 rpm (10,000 g) for 5 minutes, the supernatants were removed, and then each was resuspended in 50 μL LB. The two strains were mixed together in microcentrifuge tubes and each 100 μL mixture was plated in a puddle in the center of an LB agar plate prewarmed at 30°C for conjugation. After allowing the puddle to dry, the plates were incubated without inverting at 30°C overnight, resulting in a confluent layer of growth. The cells in the confluent growths were scraped off and suspended in 1 mL PBS. Different volumes of these suspensions were spread on VBMM + 30 μg/mL gentamicin agar plates to achieve single colony resolution and were incubated at 37°C for up to 24 hours. Single colonies were then selected and streaked onto NSLB + 15% sucrose agar plates and incubated at 30°C overnight (up to 30 hours). Single colonies were picked and checked for the desired mutation via PCR using the primers listed in S3 Table as well as confirmation of no growth with 30 μg/mL gentamicin. In some cases, the strains still grew on 30 μg/mL gentamicin after growth on NSLB + 15% sucrose agar plates, so these strains were streaked a second time (or third time if necessary) onto NSLB + 15% sucrose agar plates. Deletions were confirmed with loss of internal PCR product and presence of the external band at the appropriate sizes. To check for insertion of the *lexA(S125A)* mutation, *lexA* was amplified via PCR, and the product was sequenced with Sanger sequencing with the primers listed in S3 Table. To verify the strains were *P. aeruginosa* and not the donor *E. coli* S17-1 strains, primer pair 62 (S3 Table) was used to amplify part of *phzM* [76], which is a gene present in *P. aeruginosa* but not *E. coli*.

### Stationary-phase persister assays

*P. aeruginosa* PAO1 and MRSN 1612 strains were inoculated from -80°C, 25% glycerol stocks into 2 mL LB in 14 mL polypropylene test tubes and grown overnight at 37°C with shaking at 250 rpm. For all persister assays with *P. aeruginosa*, glycerol stocks had been prepared within 2 weeks of performing the assays by streaking a previously-prepared, unused stock onto LB agar (with antibiotics if needed for plasmid retention) and preparing fresh stocks from single colonies. After 16 hours of growth in LB, OD_600_ was measured, and the required volume to achieve OD_600_ = 0.01 in a 25 mL culture was calculated for each culture. The calculated volumes were added to 1.7 mL microcentrifuge tubes and spun down for 3 minutes at 14,800 rpm. Supernatants were removed and cell pellets were resuspended in 300 μL MOPS + 15 mM succinate. Cell suspensions were inoculated into 250 mL baffled flasks containing 25 mL MOPS + 15 mM succinate, and 300 μL were removed to measure OD_600_. Flasks were incubated at 37°C with shaking at 250 rpm for 24 hours. Persister assays were performed in MOPS minimal media because MOPS buffer contains the divalent metal ion manganese that *in vitro* studies have shown is required for LigD activity [36,66,77]. After 24 hours, flasks were removed from the shaker, and 510 μL were removed from each flask and added to 1.7 mL microcentrifuge tubes. Two-hundred and fifty μL of 1 mg/mL CIP or 200 μL of 5 mg/mL LEV were added to each flask, and flasks were returned to the incubator at 37°C with shaking at 250 rpm. This time was recorded as t = 0 hours of treatment. For solvent control treatment, an equal volume of the respective solvent instead of CIP or LEV was added to each flask. Ten μL of each 510 μL sample were used to measure OD_600_ of the cultures. Then, the remaining 500 μL of samples were washed in PBS 3 times by centrifugation for 3 minutes at 14,800 rpm, removal of 450 μL of supernatant, addition of 450 μL of PBS, and resuspension of the cell pellet. Twenty-five μL of washed samples were added to 225 μL of PBS in round-bottom 96-well plates, and 10-fold serial dilutions were performed using the same volumes. Two hundred μL of dilutions were plated onto LB agar plates and allowed to dry. At t = 1, 3, 5, and 7 hours, 500 μL samples were removed, washed 3 times in PBS to reduce the FQ concentration to below the MIC of all *P. aeruginosa* strains (MICs shown in S1 Table), serially diluted, and plated following the same steps as for t = 0 hours. After plating of all samples, plates were incubated at 37°C for 36 hours, after which CFUs were enumerated for dilutions that contained ~10 colonies or greater. Two hundred μL spots were plated and incubated for 36 hours for all strains so that mutants that formed smaller colonies (Δ*recA* and Δ*recB*) had sufficient time to grow and form visible colonies.

For *E. coli* MG1655, the same persister assay protocol was performed with some modifications. *E. coli* MG1655 WT grows very slowly when inoculated from a 16-hour growth in LB into the minimal media used here at OD_600_ ~ 0.01 (S4G Fig). Therefore, *E. coli* MG1655 strains were instead inoculated from -80°C, 25% glycerol stocks into 2 mL LB in 14 mL polypropylene test tubes and grown for 4 hours at 37°C with shaking at 250 rpm. Twenty μL of those pregrowth cultures were inoculated into 2 mL MOPS + 15 mM succinate in 14 mL polypropylene test tubes, and those cultures were incubated overnight at 37°C with shaking at 250 rpm. After 16 hours, those cultures were used to inoculate 250 mL baffled flasks containing 25 mL MOPS + 15 mM succinate following the same steps as *P. aeruginosa* strains. *E. coli* MG1655 grows more slowly than *P. aeruginosa* PAO1 in this media and reaches stationary-phase 4 hours later (S4A–S4D Fig). To account for this difference, *E. coli* cultures were incubated for 28 hours at 37°C with shaking at 250 rpm prior to treatment with CIP. After 28 hours, 1 mL samples were removed from each flask and added to 1.7 mL microcentrifuge tubes, and 10 μL were removed from each flask to measure OD_600_. Cultures were treated with CIP or solvent as described for *P. aeruginosa*. One mL samples were washed 3 times in PBS by centrifugation for 3 minutes at 14,800 rpm, removal of 950 μL of supernatant, addition of 950 μL of PBS, and resuspension of the cell pellet. This wash protocol ensured that the CIP concentration in the washed samples was below the MIC for all MG1655 strains after the third wash (MICs shown in S1 Table). Samples were serially diluted and plated following the same steps as *P. aeruginosa* strains. At t = 1, 3, 5, and 7 hours, 1 mL samples were removed, and samples were washed 3 times in PBS, serially diluted, and plated following the same steps as t = 0 hours. After plating of all samples, plates were incubated at 37°C for 36 hours, after which CFUs were enumerated for dilutions that contained at least 10 colonies.

### Exponential-phase persister assays

*P. aeruginosa* PAO1 and MRSN 1612 strains were grown from stocks and inoculated into 250 mL baffled flasks containing 25 mL MOPS + 15 mM succinate following the same steps as for stationary-phase assays. Flasks were incubated at 37°C with shaking at 250 rpm until the cultures reached mid-exponential phase (OD_600_ ~ 0.15-0.25). At that time, 1.4 mL samples were removed from each flask and added to 1.7 mL microcentrifuge tubes, and 300 μL were removed from the flasks to measure OD_600_ immediately before treatment. Two-hundred and fifty μL of 1 mg/mL CIP, 200 μL of 5 mg/mL LEV, or an equal volume of the respective solvent (instead of the FQ) were added to each flask, which were then returned to the incubator at 37°C with shaking at 250 rpm. This was recorded as t = 0 hours of treatment. Samples were pelleted at 14,800 rpm, and 1.3 mL of the supernatant were removed. Nine-hundred μL of PBS were added and the cell pellets were resuspended. The samples were washed twice more with 900 μL of PBS to reduce the FQ concentration to below the MIC of all *P. aeruginosa* strains (MICs shown in S1 Table). After the final wash, samples were pelleted for 3 minutes at 14,800 rpm, 720 μL were removed from the 1 mL sample, and the cell pellets were resuspended in the remining 280 μL, resulting in a 5-fold concentration of the original 1.4 mL sample. Samples were serially diluted and plated following the same protocol as for stationary-phase assays. At t = 1, 3, 5, and 7 hours, 1.4 mL samples were removed from each culture and washed, concentrated, serially diluted, and plated following the same steps as for t = 0 hours. When needed due to low survival, 200 μL of the 5-fold concentrated sample (no dilution) were also plated on LB agar plates. Spots were allowed to dry, and after plating of all samples, plates were incubated at 37°C for 36 hours, after which CFUs were enumerated.

Exponential-phase persister assays with *E. coli* were performed similarly to *P. aeruginosa*, but with some modifications. *E. coli* MG1655 strains were grown and inoculated into 250 mL baffled flasks containing 25 mL MOPS + 15 mM succinate following the same protocol as stationary-phase persister assays. Flasks were incubated at 37°C with shaking at 250 rpm until the cultures reached mid-exponential phase (OD_600_ ~ 0.15-0.25). At that time, samples were removed, OD_600_ was measured, and the cultures were treated with CIP or solvent following the same steps as for *P. aeruginosa*. The 1.4 mL samples were spun down at 14,800 rpm, and 1.35 mL of the supernatant were removed. Nine-hundred and fifty μL of PBS were added to each tube and the cell pellets were resuspended. The samples were washed twice more with 950 μL of PBS to ensure the CIP concentrations in the washed samples were below the MICs of all *E. coli* strains (MICs shown in S1 Table). After the third wash, samples were concentrated 5-fold, serially diluted, and plated following the same steps as for *P. aeruginosa*. At t = 1, 3, 5, and 7 hours, 1.4 mL samples were removed, washed, concentrated, serially diluted, and plated following the same steps as for t = 0 hours. For strains and time points with low survival levels, 200 μL of the 5-fold concentrated samples were plated as well. Plates were allowed to dry, and after plating of all samples, plates were incubated at 37°C for 36 hours, after which CFUs were enumerated.

### Culturability and heritability assessment

As shown in S11A Fig, WT *P. aeruginosa* PAO1 strains harboring pGL11 or pGL12 were inoculated from -80°C, 25% glycerol stocks into 2 mL LB. Gentamicin at a concentration of 30 μg/mL was included in media, buffers, and plates at all stages for plasmid retention. Overnight cultures were grown and used to inoculate 25 mL MOPS + 15 mM succinate in 250 mL baffled flasks following the same protocol as for persister assays, and flasks were incubated at 37°C with shaking at 250 rpm for 4 hours. After 4 hours of growth, the required volume of a 10 mM IPTG stock (diluted from the prepared 1 M IPTG stock) was added to each flask to achieve the desired IPTG concentration (ranging from 0-100 μM IPTG), and cultures were returned to the shaker to incubate for an additional 20 hours (for a total of 24 hours of incubation). Then, following the same protocol as t = 0 hours in *P. aeruginosa* stationary-phase persister assays, samples were removed from each flask, OD_600_ was measured, and samples were washed, serially diluted, and plated. Serial dilutions were plated onto LB agar plates containing the same concentration of IPTG as the flask culture, and plates were incubated for 36 hours to enumerate colonies. Relative culturability was calculated as the CFU/mL count after 36 hours of incubation for each induction concentration divided by the CFU/mL count for the culture that was not induced with IPTG on the same day.

To examine the heritability of culturability of surviving cells after the first round of 100 μM IPTG induction, colonies were harvested from the 100 μM IPTG agar plate after 36 hours of incubation by adding 1 mL LB + 30 μg/mL gentamicin to the plate. LB was swirled around the plate until turbid, and then 30 μL of this were added to 3 mL LB + 30 μg/mL gentamicin in a test tube. These cultures were incubated at 37°C with shaking at 250 rpm until turbid and then stocked in 25% glycerol and stored at -80°C. A second round of the culturability assay was performed identically to the first round described above, where each harvested stock was used to inoculate two flasks – one flask was induced with 100 μM IPTG and one flask was not induced.

### Persister assay with induced protein expression

WT *P. aeruginosa* PAO1 strains harboring pGL11 or pGL12 were grown to stationary-phase in 25 mL MOPS + 15 mM succinate in 250 mL baffled flasks following the same steps as for *P. aeruginosa* stationary-phase persister assays. Gentamicin at a concentration of 30 μg/mL was included in media, buffers, and plates at all stages for plasmid retention. After 24 hours, samples were removed, cultures were treated with CIP or solvent, and samples were washed and serially diluted following the same steps as for *P. aeruginosa* stationary-phase persister assays at t = 0 hours. Fifty μL of serial dilutions were plated onto filters (Supor 0.2 μm 47 mm S-Pack filters, Item #66234, Pall Corporation) on LB agar plates containing 100 μM IPTG and allowed to dry. As soon as the spots were dry, plates were incubated at 37°C for 2 hours. After 2 hours, filters were aseptically transferred using 70% ethanol- and flame-sterilized forceps to prewarmed LB agar plates without IPTG, and plates were incubated for an additional 18–22 hours to achieve a total incubation time of 20–24 hours. At t = 7 hours, 500 μL samples were removed, and these samples were washed, serially diluted, plated, and incubated following the same protocol as t = 0 hours. CFUs were enumerated for dilutions that contained at least 10 colonies.

### Statistical analysis

Three or more independent replicates were performed for each experiment, unless otherwise indicated. Data points represent means of replicates, and error bars represent standard errors of the means. Significance for persister assay survival fractions with >2 comparisons was assessed using one-way ANOVA with post-hoc Tukey tests on log-transformed t = 7 hour survival fractions and a p-value threshold of 0.05. Significance for persister assay survival fractions with 2 comparisons was assessed using two-tailed t-tests with unequal variances on log-transformed t = 7 hour survival fractions and a p-value threshold of 0.05. Significance for culturability assays with different IPTG concentrations for protein induction (Fig 4A) was assessed using two-tailed t-tests with unequal variances on log-transformed relative CFU/mL to compare relative culturability at each IPTG concentration to relative culturability with no IPTG induction. To account for multiple comparisons, a Bonferroni correction was applied to the p-value threshold, where the corrected p-value threshold was 0.05/*n*, and *n* is the total number of comparisons. Significance for culturability assays examining heritability of culturability (Fig 4B) was assessed using two-tailed t-tests with unequal variances on log-transformed relative CFU/mL and a p-value threshold of 0.05 to compare relative culturability of the first round to relative culturability of the second round of IPTG induction. Significant differences in persistence were assessed after 7 hours of treatment because by that time, the second phase of killing was readily observable, and enough time had elapsed such that normal cell contributions to CFU measurements would be negligible. For example, from Fig 1 the initial death rate for PAO1 treated with CIP in stationary- and exponential- phases were -2.86 ± 0.19 h^-1^ and -9.08 ± 0.82 h^-1^, respectively. Casting those metrics into one used to quantify tolerance, MDK_99_ (minimum duration to kill 99% of the population), we observed that the MDK_99_ of normal cells in stationary- and exponential-phase cultures were 1.71 ± 0.12 h and 0.526 ± 0.051 h, respectively. Therefore, by 7 hours of treatment the survival fractions of normal cells in stationary- and exponential- phase populations would be 2.04 x 10^-9^ and 2.50 x 10^-28^, respectively, which are both far below the survival measurements at that time and thereby establish that normal cells contributed negligibly to CFU measurements at that time.

Additional methods for this study can be found in the Supporting Information Methods (S1 Methods).

## Supporting information

S1 Methods Additional methods used in this study. (PDF)

S1 Fig *P. aeruginosa* persister survival to CIP treatment is not inherited. *P. aeruginosa* PAO1 WT was grown in MOPS minimal media with succinate to **(A and B)** stationary-phase or **(C and D)** OD_600_ ~ 0.2 and then treated with **(A and C)** 10 μg/mL CIP or **(B and D)** solvent control. Samples were taken at the indicated time points, washed, and plated on LB agar. Colonies that had survived 7 hours of CIP treatment (persister-derived population) were harvested from plates and stocked. Those persister-derived populations were then grown and treated with CIP or solvent control following the same protocol as the first round of treatment. Data points reflect the means of at least 3 biological replicates. Error bars indicate standard errors of the means. Statistical significance (p ≤ 0.05) was assessed using two-tailed t-tests with unequal variances on log-transformed CIP survival fractions at each time point. No statistical significance was found.
(PDF)

S2 Fig Complementation of Δ*recA* and Δ*recB* in *P. aeruginosa* restores stationary-phase survival during CIP treatment. *P. aeruginosa* PAO1 strains were grown in MOPS minimal media with succinate for 24 hours and then treated with **(A and B)** 10 μg/mL CIP or **(C and D)** solvent control. Samples were taken at the indicated time points, washed, and plated on LB agar for CFU enumeration. Data points reflect the means of at least 3 biological replicates. Error bars indicate standard errors of the means. One-way ANOVA with post-hoc Tukey tests were performed on log-transformed survival fractions after 7 hours of CIP treatment to assess significance. *Asterisks denote statistical significance (p ≤ 0.05), where survival fractions of Δ*recA* and Δ*recA* P_*recA*_-empty were significantly different from those of WT and Δ*recA* P_*recA*_-*recA* but not each other, and those of Δ*recA* P_*recA*_-*recA* were also significantly different from those of WT. Survival fractions of Δ*recB* and Δ*recB* P_*recB*_-empty were significantly different from those of WT and Δ*recB* P_*recB*_-*recB* but not each other, and those of Δ*recB* P_*recB*_-*recB* were not significantly different from those of WT.
(PDF)

S3 Fig *P. aeruginosa* and *E. coli* survival levels during treatment with solvent used to prepare CIP. **(A and C)** *P. aeruginosa* PAO1 strains and **(B and D)** *E. coli* MG1655 strains were grown in MOPS minimal media with succinate to **(A and B)** stationary-phase or **(C and D)** OD_600_ ~ 0.2 and then treated with the solvent used to prepare CIP. Samples were taken at the indicated time points, washed, and plated on LB agar for CFU enumeration. Data points reflect the means of at least 3 biological replicates. Error bars indicate standard errors of the means.
(PDF)

S4 Fig Growth assays indicate that some mutants of *P. aeruginosa* and *E. coli* grow more slowly than WT. **(A and B)** *P. aeruginosa* PAO1 strains and **(C and D)** *E. coli* MG1655 strains were inoculated from overnight cultures into MOPS minimal media with succinate to OD_600_ ~ 0.01. **(E and F)** *E. coli* MG1655 WT was inoculated from overnight cultures into MOPS minimal media with succinate or glucose to OD_600_ ~ 0.01. **(C-F)** *E. coli* were grown first for 4 hours in LB and then overnight in minimal media before inoculating minimal media to OD_600_ ~ 0.01. **(G)** *E. coli* were either grown with a pregrowth in LB followed by overnight growth in MOPS minimal media with succinate (circles on solid line) or grown overnight in LB (triangles on dashed line) prior to inoculation into MOPS minimal media with succinate to OD_600_ ~ 0.01. **(A, C, E, and G)** OD_600_ were measured at the indicated time points. **(B, D, and F)** Growth rates were calculated using a linear regression with ln(OD_600_) values for 3 sequential time points and are plotted at the last time point included in each linear regression. Entrance into stationary-phase was defined as the time point at which the growth rate begins to monotonically decrease toward zero and the R^2^ from the regression was less than 0.98 (indicated by arrows). Data points reflect the means of at least 3 biological replicates. Error bars indicate standard errors of the means.
(PDF)

S5 Fig Δ*recA*, Δ*recB*, and *lexA3* exhibit reduced survival in stationary-phase compared to WT when the period of time in stationary-phase is matched to that of WT. **(A-D)** *P. aeruginosa* PAO1 **(A and C)** Δ*recA* or **(B and D)** Δ*recB* were grown to stationary-phase in MOPS minimal media with succinate for 24 hours (standard incubation) or for an extended period (25 and 29 hours, respectively) to match the time in stationary-phase between WT and mutants (WT was grown for 24 hours). **(E-J)** *E. coli* MG1655 **(E and G)** Δ*recA*, **(F and H)** Δ*recB*, or **(I and J)** *lexA3* were grown to stationary-phase in MOPS minimal media with succinate for 28 hours (standard incubation) or for an extended period (29, 30, and 29 hours, respectively) to match the time in stationary-phase between WT and mutants (WT was grown for 28 hours). After the specified incubations, cultures were treated with **(A, B, E, F, and I)** 10 μg/mL CIP or **(C, D, G, H, and J)** solvent control. Samples were taken at the indicated time points, washed, and plated on LB agar for CFU enumeration. Data points reflect the means of at least 3 biological replicates. Error bars indicate standard errors of the means. One-way ANOVA with post-hoc Tukey tests were performed on log-transformed survival fractions after 7 hours of CIP treatment to assess significance. *Asterisks denote statistical significance (p ≤ 0.05). **(A, E, and I)** Survival fractions for each strain with standard and extended incubation were significantly different from those of WT but not each other. **(B and F)** Survival fractions of Δ*recB* with standard and extended incubations were significantly different from those of WT and from each other.
(PDF)

S6 Fig Treatment of *P. aeruginosa* strains that have reduced CIP MICs at 40-fold MIC shows similar results as treatment with 10 μg/mL CIP. *P. aeruginosa* PAO1 WT and mutants with reduced MICs compared to WT were grown to stationary-phase (24 h) in MOPS minimal media with succinate and then treated with 10 μg/mL CIP (40-fold MIC of WT, see S1 Table). During each replicate, each mutant was also treated with CIP at 40-fold MIC. **(A)** WT and Δ*recA* were treated with 10 μg/mL CIP, and Δ*recA* was also treated with 2 μg/mL CIP. **(B)** WT and Δ*recB* were treated with 10 μg/mL CIP, and Δ*recB* was also treated with 1.25 μg/mL CIP. **(C)** WT and *lexA(S125A)* were treated with 10 μg/mL CIP, and *lexA(S125A)* was also treated with 2.5 μg/mL CIP. Samples were taken at the indicated time points, washed, and plated on LB agar for CFU enumeration. Data points reflect the means of 3 biological replicates. Error bars indicate standard errors of the means. One-way ANOVA with post-hoc Tukey tests were performed on log-transformed survival fractions after 7 hours of treatment to assess significance. *Asterisks denote statistical significance (p ≤ 0.05), where **(A)** survival fractions of WT, Δ*recA* + 10 μg/mL CIP, and Δ*recA* + 2 μg/mL CIP are all significantly different from each other; **(B)** survival fractions of Δ*recB* + 10 μg/mL CIP and Δ*recB* + 1.25 μg/mL CIP are significantly different from those of WT but not each other; and **(C)** survival fractions of WT, *lexA(S125A)* + 10 μg/mL CIP, and *lexA(S125A)* + 2.5 μg/mL CIP are all significantly different from each other.
(PDF)

S7 Fig Stationary-phase *E. coli* grown in minimal media with glucose exhibits high tolerance to CIP. *E. coli* MG1655 WT was grown in MOPS minimal media with glucose for 26 hours and then treated with **(A)** 10 μg/mL CIP or **(B)** solvent control. Immediately before treatment (t = 0 h) and at t = 1, 3, 5, and 7 h, samples were taken, washed, and plated on LB agar. Data points reflect the means of 3 biological replicates. Error bars indicate standard errors of the means.
(PDF)

S8 Fig Fluorescent reporter demonstrates that PAO1 *lexA(S125A)* has attenuated activation of the SOS response. *P. aeruginosa* PAO1 **(A)** WT and **(B)** *lexA(S125A)* expressing pGL15 (SOS reporter) were grown in LB to exponential-phase and then treated with 10 μg/mL CIP. Immediately before treatment and then after 30 minutes and 60 minutes, samples were removed and fixed. **(A and B)** Fluorescence distribution was measured using flow cytometry. Histograms are representative of 3 biological replicates. **(C)** The percentage of the population that was GFP negative at each time point was quantified by gating 99% of the population at t = 0 min and identifying the percentage of the population within this gate at each subsequent time point from the same experiment for each strain. Data reflect the means of 3 biological replicates. Error bars indicate standard errors of the means. Two-tailed t-tests with unequal variances were performed to compare the percentages of the populations of WT and *lexA(S125A)* that were GFP negative at 30 and 60 minutes. *Asterisks denote statistical significance (p ≤ 0.05).
(PDF)

S9 Fig Loss of NHEJ and HR results in the same survival levels as loss of HR alone. **(A and B)** *P. aeruginosa* PAO1 Δ*recA* and Δ*recA* Δ*ku* were grown to **(A)** stationary-phase (24 h) or **(B)** OD_600_ ~ 0.2 in MOPS minimal media with succinate and then treated with 10 μg/mL CIP. **(C and D)** *P. aeruginosa* PAO1 WT and Δ*recA* Δ*ku* were grown for 24 hours or for an extended incubation of 25 hours (labeled Δ*recA* Δ*ku* extended) to match the time of Δ*recA* Δ*ku* cultures in stationary-phase to that of WT prior to treatment. After the specified incubation, cultures were treated with **(C)** 10 μg/mL CIP or **(D)** solvent control. Samples were taken at the indicated time points, washed, and plated on LB agar to quantify survivors. Data points reflect the means of at least 3 biological replicates. Error bars indicate standard errors of the means. **(A and B)** Two-tailed t-tests with unequal variances were performed on log-transformed survival fractions after 7 hours of treatment to assess significance. No statistical significance was found. **(C)** One-way ANOVA with post-hoc Tukey tests were performed on log-transformed survival fractions after 7 hours of treatment to assess significance. *Asterisk denotes statistical significance (p ≤ 0.05) with respect to WT.
(PDF)

S10 Fig IPTG induction results in overexpression of Ku and LigD from synthetic operon in *P. aeruginosa*. *P. aeruginosa* PAO1 WT expressing pGL10 (P_*tac*_ empty), pGL11 (P_*tac*_*-sfgfp*), or pGL12 (P_*tac*_*-ku-ligD*) were grown to exponential-phase in LB and then treated with 1 mM IPTG for 4 hours prior to preparation of protein lysates. **(A)** SDS-PAGE gel. Lanes 1 and 5: Protein ladder. Lane 2: WT + pGL10 (P_*tac*_ empty). Lane 3: WT + pGL11 (P_*tac*_*-sfgfp*). Lane 4: WT + pGL12 (P_*tac*_*-ku-ligD*). Red arrows indicate bands suspected to contain LigD (94 kDa) and Ku (32.8 kDa) based on size. **(B and C)** Samples suspected to contain LigD and Ku based on size from lane 4 were cut and analyzed via mass spectrometry. Protein sequence coverage for **(B)** LigD and **(C)** Ku samples were 85% and 91%, respectively. Yellow highlight indicates identified peptides.
(PDF)

S11 Fig Assessing how timing of induction of Ku and LigD expression in *P. aeruginosa* impacts culturability. **(A and B)** Schematics demonstrating different methods of inducing Ku and LigD expression in *P. aeruginosa*. **(A)** *P. aeruginosa* PAO1 WT harboring pGL11 or pGL12 were grown for 4 hours in MOPS minimal media with succinate and then induced with IPTG at a range of concentrations. Cultures were incubated for an additional 20 hours, and then samples were removed, washed, and plated on LB agar plates containing IPTG at the same concentration as the flask cultures. Created in BioRender. Leon, G. (2025) https://BioRender.com/y74h037 **(B)** *P. aeruginosa* PAO1 WT harboring pGL11 or pGL12 were grown for 24 hours in MOPS minimal media with succinate. Cultures were incubated for an additional 7 hours (corresponds to the time of persister assays) without treatment, and then samples were removed, washed, and plated on filters on LB agar plates. After two hours of incubation, filters were transferred to a second set of LB agar plates and incubated for an additional 18–22 hours. For method I, no IPTG was included to serve as a basis for comparison. For method II, 100 μM IPTG was included only in the first set of LB agar plates. For method III, after 22 hours of initial growth, 100 μM IPTG was added to the cultures. After an additional 9 hours of incubation, samples were plated onto filters on the first set of LB agar plates, which contained 100 μM IPTG. The filters were later transferred to IPTG-free LB agar plates. Created in BioRender. Leon, G. (2025) https://BioRender.com/f26j739 **(C)** The protocol described in **(B)** was performed, and culturability was measured as CFU/mL for each induction condition (IPTG on first plate only or IPTG in flask culture and on first plate) relative to CFU/mL with no induction for the same strain during the same experiment. Data reflect the means of at least 3 biological replicates. Error bars indicate standard errors of the means. Two-tailed t-tests with unequal variances were performed on log-transformed relative CFU/mL to assess significance. *Asterisk denotes statistical significance (defined as p ≤ 0.05) with respect to no induction (relative culturability of 1).
(PDF)

S12 Fig *P. aeruginosa* survival levels during treatment with solvent used to prepare LEV. *P. aeruginosa* PAO1 strains were grown in MOPS minimal media with succinate to **(A)** stationary-phase or **(B)** OD_600_ ~ 0.2 and then treated with the solvent used to prepare LEV. Samples were taken at the indicated time points, washed, and plated on LB agar for CFU enumeration. Data points reflect the means of at least 2 biological replicates. Error bars indicate standard errors of the means.
(PDF)

S1 Table MICs for *P. aeruginosa* and *E. coli.* CIP and LEV MICs were measured with E-test strips (Liofilchem). MICs represent the range observed for at least 3 biological replicates.
(PDF)

S2 Table Strains and plasmids used in this study. (PDF)

S3 Table Primers used in this study for cloning, strain construction, and plasmid and strain verification. (PDF)
