# Supplementary material for: Conservation and divergence of ciprofloxacin persister survival mechanisms between Pseudomonas aeruginosa and Escherichia coli
Source: PLoS Genet. 2025 Sep 2;21(9):e1011840. doi: 10.1371/journal.pgen.1011840 (PMC12413089; doi:10.1371/journal.pgen.1011840)
Supplement: S1 Methods — (PDF) [file pgen.1011840.s001.pdf]

S1 Methods

Plasmid construction

pBBR1MCS-2 was a gift from Kenneth Peterson (Addgene plasmid #85168; http://n2t.net/addgene:85168; RRID: Addgene 85168). Plasmids pGL01, pGL02, and pGL05 were constructed via restriction digestion with the primers and restriction enzymes described in S2 Table and S3 Table, and ligation was performed with Quick Ligase (New England Biolabs, Inc.). Plasmid pGL08 was constructed via site-directed mutagenesis with Q5 Site-Directed Mutagenesis Kit (New England Biolabs, Inc.) as described in S2 Table and S3 Table. All other plasmids were constructed using Gibson Assembly with Gibson Assembly Master Mix (New England Biolabs, Inc.) as described in S2 Table and S3 Table. All PCRs amplifying genomic DNA or plasmid DNA were performed using Phusion High-Fidelity DNA polymerase (New England Biolabs, Inc.) or Q5 High-Fidelity DNA polymerase (New England Biolabs, Inc.). Colony PCRs to check constructs were performed using Taq DNA polymerase (New England Biolabs, Inc.). Primer pairs for plasmid construction and verification via PCR and/or Sanger sequencing are shown in S3 Table.

Complementation strain construction

For complementation in *P. aeruginosa* PAO1, the *recA* and *recB* genes were inserted onto the chromosome of the Δ*recA* and Δ*recB* strains, respectively, under the control of their native promoters using the Tn7 transposition system developed by Choi and colleagues (1), generating strains Δ*recA* P_{recA}-*recA* and Δ*recB* P_{recB}-*recB*, respectively. As controls, the native promoters without the *recA* and *recB* genes were inserted, generating strains Δ*recA* P_{recA}-empty and Δ*recB* P_{recB}-empty, respectively. For Tn7 transposition, 50 ng of pTNS2 (2) together with 50 ng of either pGL16 or pGL17 were simultaneously transformed into PAO1 Δ*recA* (for Δ*recA* P_{recA}-

empty and Δ*recA* P_{recA}-*recA*, respectively), and 50 ng of pTNS2 together with 50 ng of either pGL18 or pGL19 were simultaneously transformed into PAO1 Δ*recB* (for Δ*recB* P_{recB}-empty and Δ*recB* P_{recB}-*recB*, respectively). Outgrowths after transformation were plated onto LB agar plates containing 30 μg/mL gentamicin for selection. Colonies were checked via PCR and/or Sanger sequencing for integration of the mini-Tn7 element at the *att*Tn7 site (downstream of the *glmS* gene) using the primer pairs shown in S3 Table. Strains were also confirmed to still have the *recA* or *recB* knockout at the native locus using primer pairs external to the knocked-out gene as described in S3 Table.

MIC assessment

Strains were grown overnight (~16 hours) in 2 mL LB from a -80°C, 25% glycerol stock at 37°C with shaking at 250 rpm. After overnight growth, 1 mL of each culture was pelleted for 3 minutes at 14,800 rpm and resuspended in autoclaved 0.85% NaCl. These cell suspensions were diluted to OD_{600} ~ 0.2 (McFarland Turbidity standard of 0.5, or ~1-2x10^8 cells/mL) in 1 mL autoclaved 0.85% NaCl in a 14 mL test tube. A sterile swab was then used to distribute each suspension over a Mueller Hinton II agar (prepared according to manufacturer's instructions, BD) plate with a depth of ~4 mm by soaking the sterile swab in the cell suspension and spreading evenly across the entire surface of the plate three times, rotating the plate ~60° between each application. After allowing the plates to completely dry for at least 15 minutes, a CIP E-test strip (Liofilchem MTS Ciprofloxacin 0.002-32 μg/mL) or LEV E-test strip (Liofilchem MTS Levofloxacin 0.002-32 μg/mL) was applied to the center of the plate using sterile tweezers. After 16-20 hours of incubation at 37°C, the MIC was read as the concentration at (or concentrations between) which the ellipse of no growth intersected the strip.

Persistence heritability

P. aeruginosa PAO1 WT persisters surviving one round of stationary-phase or exponential-phase CIP treatment were assessed for heritability of the persistence phenotype. Cells surviving the first round of CIP treatment were harvested by adding 1 mL of LB media to an LB agar plate containing only the CFUs that survived 7 hours of CIP treatment after 36 hours of incubation of the plate. LB was swirled around the plate until turbid, and then 30 μL of this suspension were inoculated into 3 mL of LB in a test tube. This culture was grown until turbid, stocked in 25% glycerol, and stored at -80°C for up to two weeks. This stock was used to perform a second stationary-phase or exponential-phase persister assay identical to the first round following the same protocols as described previously.

Growth assays

For *P. aeruginosa* growth assays, strains were grown and inoculated into 25 mL MOPS + 15 mM succinate following the same protocol as for the *P. aeruginosa* persister assays. For all *E. coli* growth assays with pregrowth, *E. coli* strains were grown and inoculated into 25 mL MOPS + 15 mM succinate or MOPS + 10 mM glucose as specified following the protocol described for the *E. coli* persister assays. MOPS + 10 mM glucose was prepared the same way as MOPS + 15 mM succinate, except glucose was added from a sterile-filtered, 1 M glucose stock solution to achieve a final concentration of 10 mM glucose. For growth assays with *E. coli* MG1655 WT without pregrowth, *E. coli* WT was grown and inoculated into 25 mL MOPS + 15 mM succinate following the same protocol as for *P. aeruginosa* growth assays. The OD_{600} of each flask culture was measured immediately after inoculation with 300 μL, and then flasks were returned to the incubator to grow at 37°C with shaking at 250 rpm. This was recorded as t = 0 hours. At each time point shown in S4 Fig growth assays, 300 μL samples were removed from each flask to

measure OD_{600}. When $OD_{600} > \sim 0.3$, samples were diluted prior to measuring OD_{600} to ensure that the measurement was within the linear range of the plate reader. The growth rates shown in S4 Fig were calculated by performing a linear regression on $\ln(OD_{600})$ for each grouping of 3 sequential time points. Entrance into stationary-phase was defined as the first time point at which both the growth rate begins a monotonic decline toward zero and the $R^2 < 0.98$ for the linear regression on $\ln(OD_{600})$.

Extended incubation persister assays

P. aeruginosa PAO1 strains Δ*recA*, Δ*recB*, and Δ*recA* Δ*ku* and *E. coli* MG1655 strains Δ*recA*, Δ*recB*, and *lexA3* grew more slowly than their respective WT strains and entered stationary-phase later (S4A-D Fig). Therefore, persister assays were performed with an extended period of incubation in 25 mL MOPS + 15 mM succinate prior to CIP or solvent treatment so that each slow-growing strain was in stationary-phase in minimal media for the same amount of time as the respective WT strain. These stationary-phase assays were performed identically to the stationary-phase assays previously described for *P. aeruginosa* and *E. coli*, except *P. aeruginosa* PAO1 Δ*recA* and Δ*recA* Δ*ku* cultures were incubated for 25 hours (+1 hour), *P. aeruginosa* Δ*recB* cultures were incubated for 29 hours (+5 hours), *E. coli* Δ*recA* and *lexA3* were incubated for 29 hours (+1 hour) and *E. coli* Δ*recB* cultures were incubated for 30 hours (+2 hours) at 37°C with shaking at 250 rpm prior to treatment. After the specified incubation times, samples were taken, cultures were treated with CIP or solvent control, and samples were washed and plated following the same protocols as described previously for the stationary-phase persister assays for each species.

Persister assays with *P. aeruginosa* treated with 40xMIC CIP

Stationary-phase persister assays with *P. aeruginosa* PAO1 Δ*recA*, Δ*recB*, and *lexA(S125A)* were performed where each strain was treated with CIP at a concentration ~40-fold higher than the MIC (S1 Table). *P. aeruginosa* PAO1 Δ*recA*, Δ*recB*, and *lexA(S125A)* were treated with 2, 1.25, and 2.5 μg/mL CIP, respectively, from 100-fold concentrated stocks that had been prepared by diluting a 5 mg/mL CIP stock in water and sterilizing with a 0.22 μm syringe filter. Persister assays were performed identically to the *P. aeruginosa* stationary-phase persister assays described in the Main methods, where 250 μL of the 100-fold concentrated stock was used to treat each strain. During each experiment, assays were simultaneously performed where each strain and WT were treated with 10 μg/mL CIP for direct comparison of persister levels.

***E. coli* stationary-phase persister assay in 10 mM glucose**

E. coli MG1655 WT was grown to stationary-phase in MOPS + 10 mM glucose and treated with 10 μg/mL CIP or solvent following the same protocol as the stationary-phase persister assays in MOPS + 15 mM succinate, except cultures were incubated for 26 hours in 25 mL MOPS + 10 mM glucose prior to treatment to match the time in stationary-phase to that of WT grown in MOPS + 15 mM succinate (S4EF Fig). Samples were taken, washed, plated, and incubated following the same protocol as the *E. coli* stationary-phase persister assays.

SOS response activation with fluorescent transcriptional reporter

P. aeruginosa PAO1 WT and *lexA(S125A)* harboring pGL15 were grown overnight in 2 mL LB from -80°C, 25% glycerol stocks at 37°C with shaking at 250 rpm for ~16 hours. Thirty μg/mL of gentamicin was included in LB at all stages for plasmid retention. After ~16 hours of growth, OD_{600} of each culture was measured and the volume required to inoculate 3 mL of LB to OD_{600} ~ 0.01 was calculated. For each strain, this volume was mixed in 300 μL LB, the sample

was used to inoculate a test tube containing 3 mL of LB, and then 300 μL were removed from the test tube to measure OD_{600}. Cultures were grown at 37°C with shaking at 250 rpm until OD_{600} ~ 0.2-0.3. When OD_{600} ~ 0.2-0.3, 500 μL were removed from each tube for fixation. The culture was then treated with 10 μg/mL CIP and returned to the shaker. This time was recorded as t = 0 minutes. Samples were fixed by centrifuging 500 μL cell samples at 14,800 rpm for 3 minutes, removing all of the supernatants, resuspending cell pellets in 4% PFA, and incubating at room temperature for 15-30 minutes. To remove PFA, fixed samples were centrifuged again, all of the supernatants were removed, and fixed cells were resuspended in 500 μL PBS. These fixed samples were stored at 4°C until flow cytometry. At t = 30 and 60 minutes of incubation with CIP, 500 μL samples were removed from each culture and fixed with 4% PFA following the same protocol as prior to treatment. For flow cytometry, all fixed samples in PBS were diluted to OD_{600} ~ 0.04 in 1 mL of PBS in 5 mL polystyrene tubes (Corning Falcon 352054) and stored at 4°C until flow cytometry. GFP fluorescence was measured on an LSRII flow cytometer (BD Biosciences) with an excitation wavelength and emission bandpass filter of 488 nm and 525/50, respectively, and ~50,000 events were collected. Cells were gated from forward scatter versus side scatter plots. Then, a gate was drawn on the GFP fluorescence histogram to capture 99% of the cell population of each strain at t = 0 min (immediately before CIP treatment). This gate was applied to each subsequent time point during a given experiment for each strain to determine the percentage of the cell population within this gate, which is the percentage of the population that was considered GFP negative.

Confirmation of protein overexpression with IPTG induction via protein gel analysis and mass spectrometry

P. aeruginosa PAO1 WT harboring pGL10, pGL11 (expresses sfGFP from P_{tac}), and pGL12 (expresses Ku and LigD from P_{tac}) were inoculated into 2 mL LB in 14 mL polypropylene test tubes from -80°C, 25% glycerol stocks and incubated at 37°C with shaking at 250 rpm for 16 hours. Thirty μg/mL of gentamicin was included in growth medias at all stages for plasmid retention. After 16 hours, OD_{600} of cultures were measured and the required volume to inoculate 2 mL of LB to OD_{600} ~ 0.01 was calculated. The required volume was mixed with LB in a 1.7 mL microcentrifuge tube to achieve a total volume of 300 μL. This inoculum was added to 2 mL LB in test tubes and mixed, 300 μL were removed to measure OD_{600}, and cultures were incubated at 37°C with shaking at 250 rpm. When cultures reached exponential-phase (OD_{600} ~ 0.05-0.10), cultures were induced with 1 mM IPTG from a 1 M IPTG stock. After 4 hours of induction, 500 μL of each culture were pelleted, supernatants were removed, and each cell pellet was resuspended in 100 μL of sterile water. These samples were then diluted with water to OD_{600} ~ 7-8 in 40 μL total volume. Thirty μL of each cell suspension were mixed with 30 μL of 2x Laemmli sample buffer (Bio-Rad Laboratories, Inc.) in 1.7 mL microcentrifuge tubes, and these samples were lysed by placing the microcentrifuge tubes in a water bath and boiling in the microwave for > 4 minutes. Twenty μL of each sample were run on a Mini-PROTEAN TGX Stain-free precast 7.5% gel (Bio-Rad Laboratories, Inc.) for 31 minutes at 200 volts. The protein gel was washed in water with shaking at 50 rpm at room temperature for 15 minutes, and then the gel was removed from the water and stained overnight (16 hours) in staining solution (50% v/v methanol, 0.05% w/v Coomassie brilliant blue R-250 (Bio-Rad), 10% v/v acetic acid in water) with shaking at 50 rpm at room temperature. After staining, the gel was

destained by boiling in water for 8 minutes in the microwave twice, where the water was changed between each wash. An image of the gel is shown in S10A Fig. For mass spectrometry of the bands suspected to contain Ku and LigD, the appropriate bands from the lane containing the sample from *P. aeruginosa* PAO1 WT harboring pGL12 were cut and stored in a microcentrifuge tube with water at 4°C until mass spectrometry was performed. In-gel digestion of protein bands using trypsin was performed as described by Shevchenko and colleagues (3). Trypsin digested samples were dried completely in a SpeedVac and resuspended with 20 µL of 0.1% formic acid pH 3 in water. Two µL (~360 ng) were injected per run using an Easy-nLC 1200 UPLC system. Samples were loaded directly onto a 45 cm long 75 µm inner diameter nano capillary column packed with 1.9 µm C18-AQ resin (Dr. Maisch, Germany) mated to metal emitter in-line with an Orbitrap Fusion Lumos (Thermo Scientific, USA). Column temperature was set at 45°C, and two-hour gradient method with 300 nL per minute flow was used. The mass spectrometer was operated in data dependent mode with the 120,000 resolution MS1 scan (positive mode, profile data type, AGC gain of 4e5, maximum injection time of 54 seconds and mass range of 375-1500 m/z) in the Orbitrap followed by HCD fragmentation in Orbitrap (30,000 resolution) with 35% collision energy. Dynamic exclusion list was invoked to exclude previously sequenced peptides for 60 s and maximum cycle time of 3 s was used. Peptides were isolated for fragmentation using quadrupole (1.2 m/z isolation window).

Raw files were searched using MSAmanda 2.0 (4) and Sequest HT algorithms (5) within the Proteome Discoverer 2.5.0 suite (Thermo Scientific, USA). Ten ppm MS1 and 0.4 Da MS2 mass tolerances were specified. Carbamidomethylation of cysteine was used as fixed modification, oxidation of methionine, deamidation of asparagine and glutamine were specified as dynamic modifications. Pyro glutamate conversion from glutamic acid and glutamine are set

as dynamic modifications at peptide N-terminus. Acetylation was specified as dynamic modification at protein N-terminus. Trypsin digestion with maximum of 2 missed cleavages were allowed. Files were searched against *P. aeruginosa* strain ATCC 15692 (organism ID 208964) database downloaded from Uniprot.org.

Scaffold (version Scaffold 5.1.0, Proteome Software Inc., Portland, OR) was used to validate MS/MS based peptide and protein identifications. Peptide identifications were accepted if they could be established at greater than 95.0% probability by the Scaffold Local FDR algorithm. Protein identifications were accepted if they could be established at greater than 99.9% probability and contained at least 2 identified peptides. Protein probabilities were assigned by the Protein Prophet algorithm (6).

Culturability of PAO1 with reduced time of protein induction

To assess the impact of shorter induction of Ku and LigD expression on *P. aeruginosa* culturability, culturability was assessed following the schematic in S11B Fig. *P. aeruginosa* PAO1 WT strains harboring pGL11 or pGL12 were grown and inoculated into 25 mL MOPS + 15 mM succinate following the same steps as described for the culturability assessment. Thirty μg/mL of gentamicin was included in all growth medias, buffers, and plates at all stages for plasmid retention. Flask cultures were incubated at 37°C with shaking at 250 rpm. One flask culture of each strain was grown without any IPTG induction for 31 hours, and a second flask culture of each strain was induced with 100 μM IPTG from a 10 mM IPTG working stock (diluted from sterile 1 M IPTG stock) after 22 hours of growth and then returned to incubate at 37°C with shaking at 250 rpm for an additional 9 hours. After 31 total hours of incubation of all flasks (to account for 24 hours of growth before t = 0 hour of a persister assay + 7 hours of treatment time during a persister assay), flasks were removed from the shaker, and 510 μL

samples were removed from each flask and added to 1.7 mL microcentrifuge tubes. Ten μL of each sample were used to measure OD_{600}, and then the samples were washed and serially diluted following the same protocol described for the persister assay with induced expression. Fifty μL of dilutions from the flask with no IPTG induction were plated onto filters (Supor 0.2 μm 47 mm S-Pack filters, Item #66234, Pall Corporation) on LB agar plates containing either no IPTG or 100 μM IPTG and were allowed to dry (conditions I and II, respectively, in S11B Fig). Fifty μL of dilutions from the flask culture that had been induced with 100 μM IPTG were plated on filters on plates containing 100 μM IPTG and were allowed to dry (condition III in S11B Fig). As soon as the spots were dry, plates were incubated at 37°C for 2 hours, and then filters were aseptically transferred to prewarmed LB agar plates with no IPTG using 70% ethanol- and flame-sterilized forceps. Plates were incubated for an additional 18-22 hours for a total incubation time of 20-24 hours. CFUs were enumerated for dilutions that contained at least 10 colonies. Relative culturability was calculated as the CFU/mL count for each induction method divided by the CFU/mL count for the culture that was not induced with IPTG on the same day.

Calculating killing parameters

To characterize killing dynamics for *P. aeruginosa* PAO1 populations treated with 10 μg/mL CIP during stationary- and exponential-phases, which exhibit biphasic killing, populations were assumed to have two subpopulations that are each characterized by exponential death, as described in Equation 1, where t represents time, x represents survival fraction at time t, k_d represents net death rate, and x_0 represents survival fraction at the initial time point.

$$x = x_0 \cdot e^{k_d \cdot t} \qquad \text{Equation 1}$$

The initial death rate was calculated using Equation 1 from the survival fractions at t = 0 hours and t = 1 hour. The MDK_{99}, which describes the minimum duration required to kill 99% of the population, was then calculated by setting $x_0 = 1$ and $x = 0.01$, which results in Equation 2.

$$MDK_{99} = \frac{\ln(0.01)}{k_d} \qquad \text{Equation 2}$$

The MDK_{99} was calculated for the initial population using the calculated initial death rate. After determining the death rate for the initial population, the estimated survival fraction of the initial population (which is majority non-persister cells) at t = 7 hours was calculated using Equation 1.

Supporting Information References

1. Choi K-H, Schweizer HP. 2006. mini-Tn7 insertion in bacteria with single attTn7 sites: example Pseudomonas aeruginosa. Nature Protocols 1:153-161.
2. Choi K-H, Gaynor JB, White KG, Lopez C, Bosio CM, Karkhoff-Schweizer RR, Schweizer HP. 2005. A Tn7-based broad-range bacterial cloning and expression system. Nature Methods 2:443-448.
3. Shevchenko A, Tomas H, Havli J, Olsen JV, Mann M. 2006. In-gel digestion for mass spectrometric characterization of proteins and proteomes. Nature Protocols 1:2856-2860.
4. Dorfer V, Pichler P, Stranzl T, Stadlmann J, Taus T, Winkler S, Mechtler K. 2014. MS Amanda, a universal identification algorithm optimized for high accuracy tandem mass spectra. J Proteome Res 13:3679-84.
5. Eng JK, McCormack AL, Yates JR. 1994. An approach to correlate tandem mass spectral data of peptides with amino acid sequences in a protein database. J Am Soc Mass Spectrom 5:976-89.
6. Nesvizhskii AI, Keller A, Kolker E, Aebersold R. 2003. A Statistical Model for Identifying Proteins by Tandem Mass Spectrometry. Analytical Chemistry 75:4646-4658.
7. Kohanski MA, Dwyer DJ, Hayete B, Lawrence CA, Collins JJ. 2007. A Common Mechanism of Cellular Death Induced by Bactericidal Antibiotics. Cell 130:797-810.
8. Lemma AS, Soto-Echevarria N, Brynildsen MP. 2022. Fluoroquinolone Persistence in Escherichia coli Requires DNA Repair despite Differing between Starving Populations. Microorganisms. 10(2):doi:10.3390/microorganisms10020286.
9. Koch MD, Black ME, Han E, Shaevitz JW, Gitai Z. 2022. Pseudomonas aeruginosa distinguishes surfaces by stiffness using retraction of type IV pili. Proceedings of the National Academy of Sciences 119:e2119434119.
10. Shen Y, Siryaporn A, Lecuyer S, Gitai Z, Stone Howard A. 2012. Flow Directs Surface-Attached Bacteria to Twitch Upstream. Biophysical Journal 103:146-151.

11. Kovach ME, Elzer PH, Hill DS, Robertson GT, Farris MA, Roop RM, 2nd, Peterson KM. 1995. Four new derivatives of the broad-host-range cloning vector pBBR1MCS, carrying different antibiotic-resistance cassettes. Gene 166:175-6.
12. Rietsch A, Vallet-Gely I, Dove SL, Mekalanos JJ. 2005. ExsE, a secreted regulator of type III secretion genes in Pseudomonas aeruginosa. Proceedings of the National Academy of Sciences 102:8006-8011.
13. Amato Stephanie M, Brynildsen Mark P. 2015. Persister Heterogeneity Arising from a Single Metabolic Stress. Current Biology 25:2090-2098.
14. Lesic B, Rahme LG. 2008. Use of the lambda Red recombinase system to rapidly generate mutants in Pseudomonas aeruginosa. BMC molecular biology 9:20-20.
15. Cherepanov PP, Wackernagel W. 1995. Gene disruption in Escherichia coli: TcR and KmR cassettes with the option of Flp-catalyzed excision of the antibiotic-resistance determinant. Gene 158:9-14.
16. Fürste JP, Pansegrau W, Frank R, Blöcker H, Scholz P, Bagdasarian M, Lanka E. 1986. Molecular cloning of the plasmid RP4 primase region in a multi-host-range tacP expression vector. Gene 48:119-131.
17. Robinson JL, Brynildsen MP. 2015. An ensemble-guided approach identifies ClpP as a major regulator of transcript levels in nitric oxide-stressed Escherichia coli. Metabolic Engineering 31:22-34.
