# Supplementary figures and images for: Conservation and divergence of ciprofloxacin persister survival mechanisms between Pseudomonas aeruginosa and Escherichia coli
Source: PLoS Genet. 2025 Sep 2;21(9):e1011840. doi: 10.1371/journal.pgen.1011840 (PMC12413089; doi:10.1371/journal.pgen.1011840)

*P. aeruginosa* (MOPS media with 15 mM succinate)

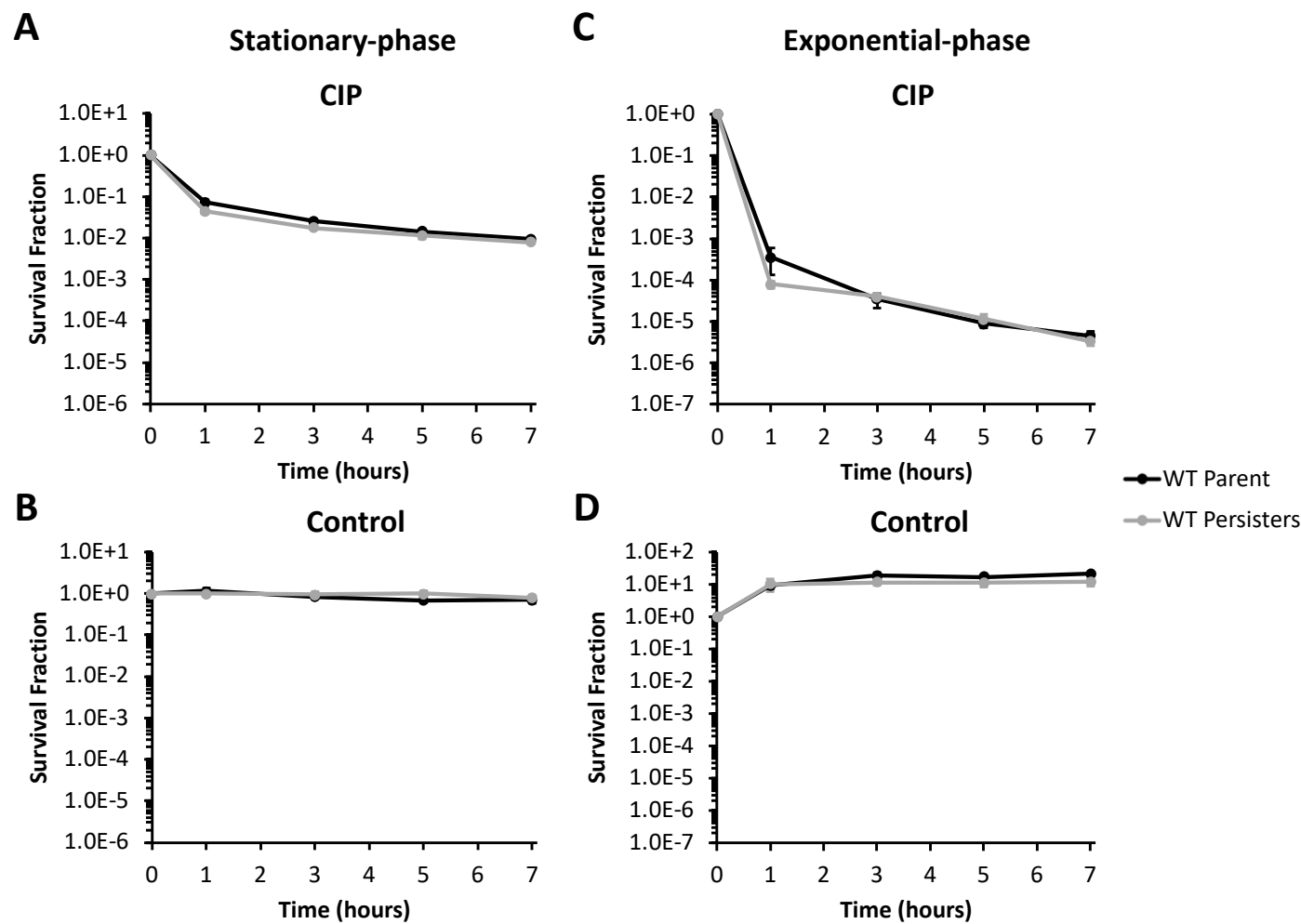

S1 Figure

Supplement: S1 Fig — P. aeruginosa PAO1 WT was grown in MOPS minimal media with succinate to (A and B) stationary-phase or (C and D) OD600 ~ 0.2 and then treated with (A and C) 10 μg/mL CIP or (B and D) solvent control. Samples were taken at the indicated time points, washed, and plated on LB agar. Colonies that had survived 7 hours of CIP treatment (persister-derived population) were harvested from plates and stocked. Those persister-derived populations were then grown and treated with CIP or solvent control following the same protocol as the first round of treatment. Data points reflect the means of at least 3 biological replicates. Error bars indicate standard errors of the means. Statistical significance (p ≤ 0.05) was assessed using two-tailed t-tests with unequal variances on log-transformed CIP survival fractions at each time point. No statistical significance was found. (PDF) [file pgen.1011840.s002.pdf]

*P. aeruginosa* (MOPS media with 15 mM succinate)

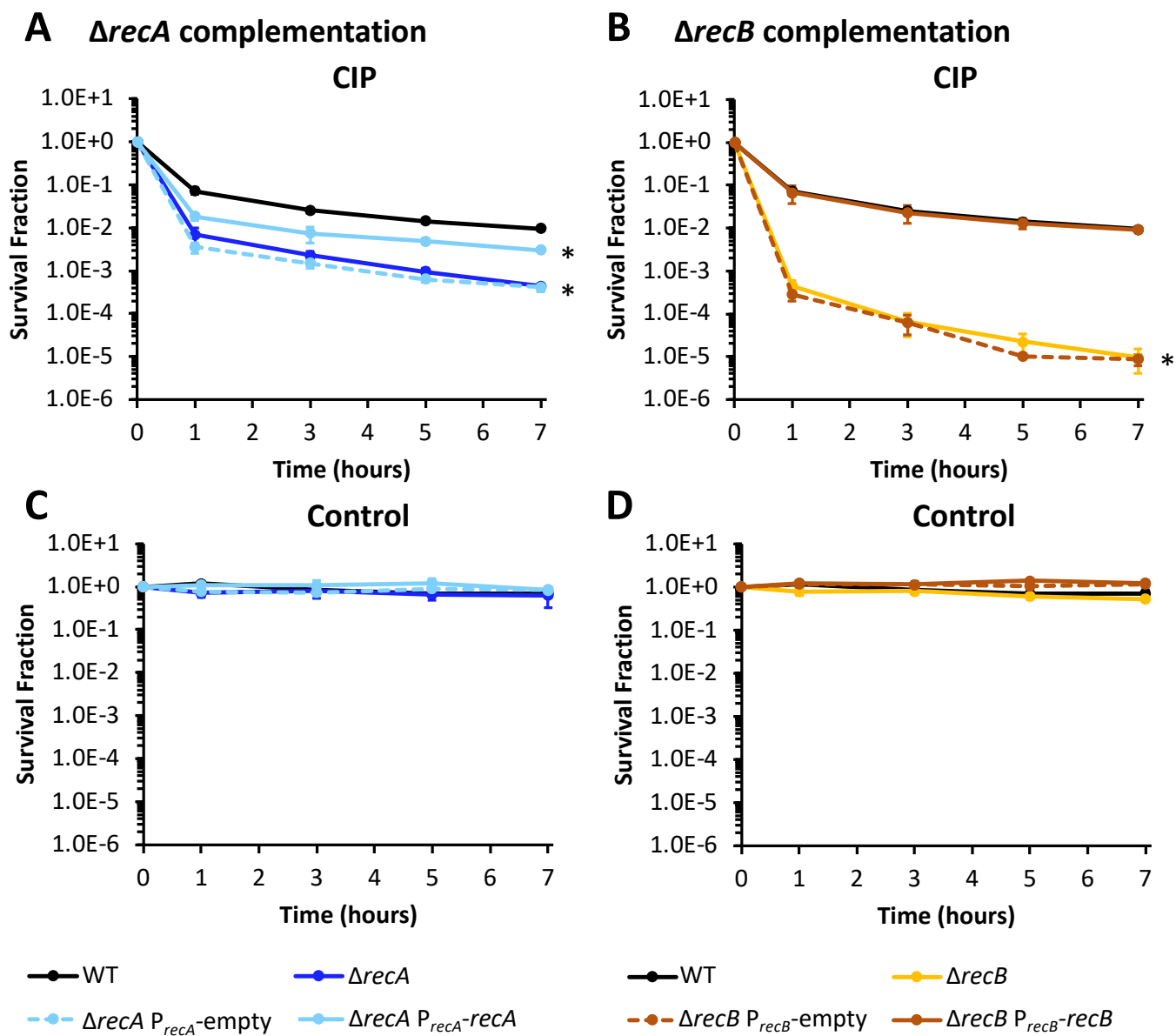

S2 Figure

Supplement: S2 Fig — P. aeruginosa PAO1 strains were grown in MOPS minimal media with succinate for 24 hours and then treated with (A and B) 10 μg/mL CIP or (C and D) solvent control. Samples were taken at the indicated time points, washed, and plated on LB agar for CFU enumeration. Data points reflect the means of at least 3 biological replicates. Error bars indicate standard errors of the means. One-way ANOVA with post-hoc Tukey tests were performed on log-transformed survival fractions after 7 hours of CIP treatment to assess significance. *Asterisks denote statistical significance (p ≤ 0.05), where survival fractions of ΔrecA and ΔrecA PrecA-empty were significantly different from those of WT and ΔrecA PrecA-recA but not each other, and those of ΔrecA PrecA-recA were also significantly different from those of WT. Survival fractions of ΔrecB and ΔrecB PrecB-empty were significantly different from those of WT and ΔrecB PrecB-recB but not each other, and those of ΔrecB PrecB-recB were not significantly different from those of WT. (PDF) [file pgen.1011840.s003.pdf]

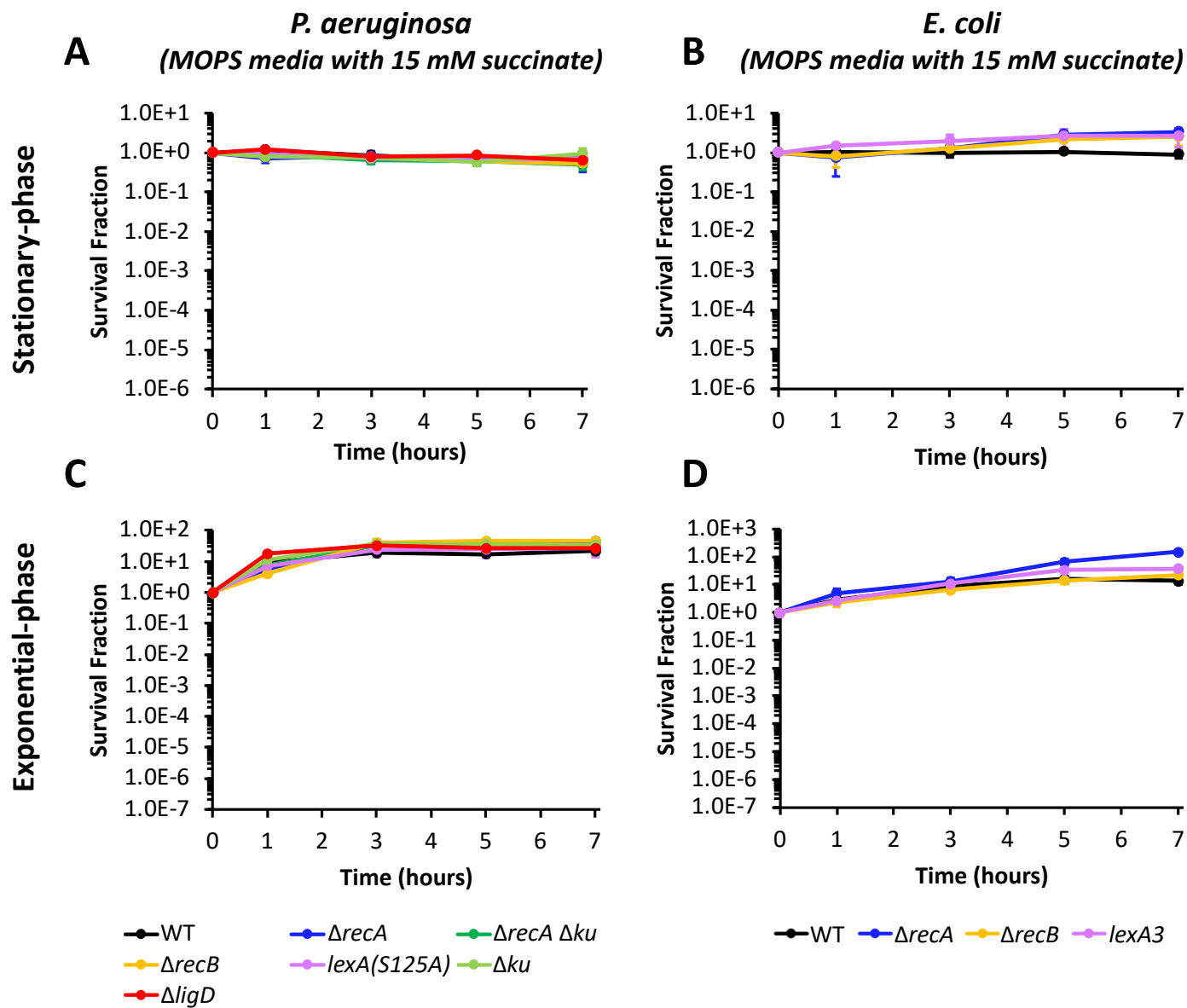

S3 Figure

Supplement: S3 Fig — (A and C) P. aeruginosa PAO1 strains and (B and D) E. coli MG1655 strains were grown in MOPS minimal media with succinate to (A and B) stationary-phase or (C and D) OD600 ~ 0.2 and then treated with the solvent used to prepare CIP. Samples were taken at the indicated time points, washed, and plated on LB agar for CFU enumeration. Data points reflect the means of at least 3 biological replicates. Error bars indicate standard errors of the means. (PDF) [file pgen.1011840.s004.pdf]

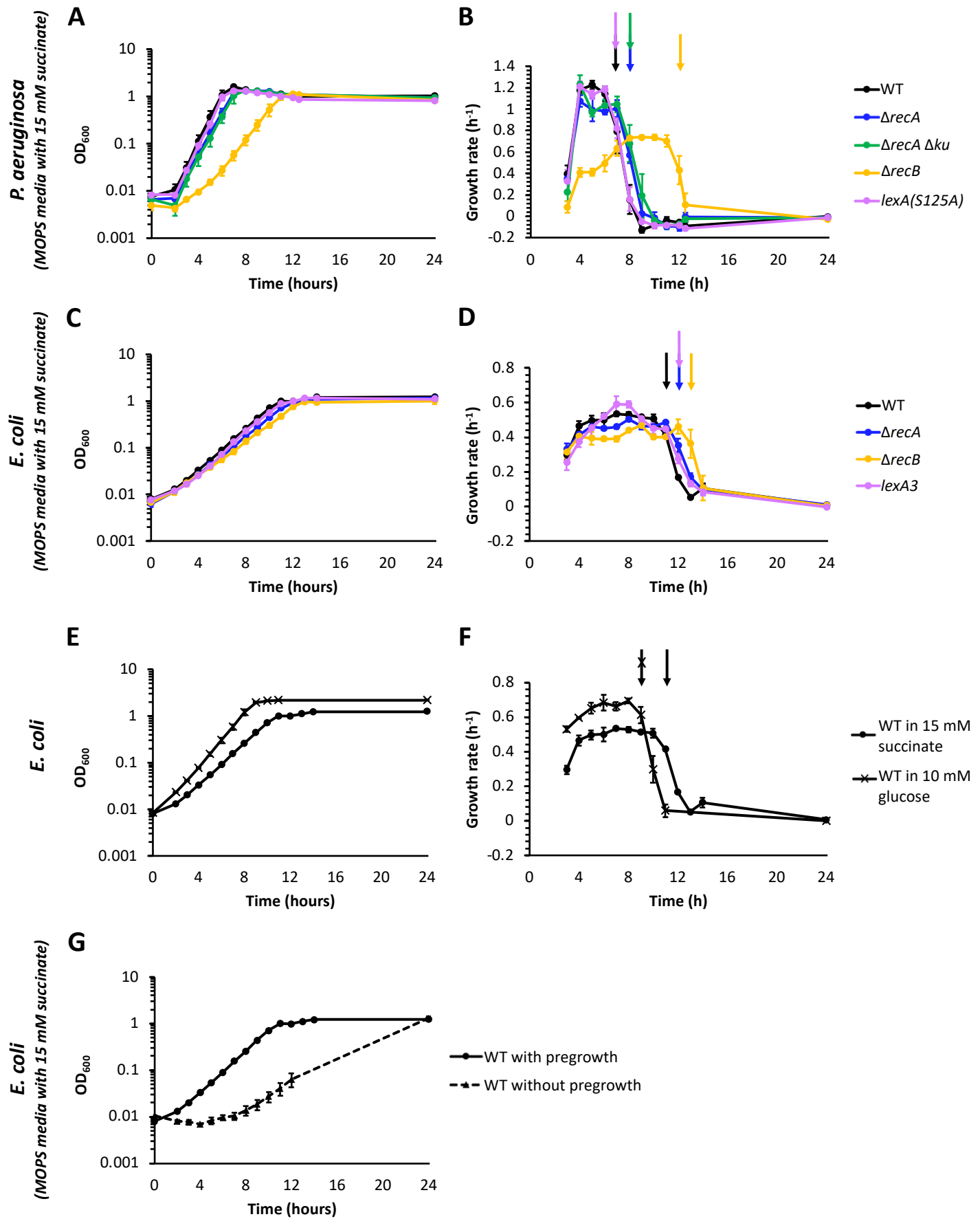

S4 Figure

Supplement: S4 Fig — (A and B) P. aeruginosa PAO1 strains and (C and D) E. coli MG1655 strains were inoculated from overnight cultures into MOPS minimal media with succinate to OD600 ~ 0.01. (E and F) E. coli MG1655 WT was inoculated from overnight cultures into MOPS minimal media with succinate or glucose to OD600 ~ 0.01. (C-F) E. coli were grown first for 4 hours in LB and then overnight in minimal media before inoculating minimal media to OD600 ~ 0.01. (G) E. coli were either grown with a pregrowth in LB followed by overnight growth in MOPS minimal media with succinate (circles on solid line) or grown overnight in LB (triangles on dashed line) prior to inoculation into MOPS minimal media with succinate to OD600 ~ 0.01. (A, C, E, and G) OD600 were measured at the indicated time points. (B, D, and F) Growth rates were calculated using a linear regression with ln(OD600) values for 3 sequential time points and are plotted at the last time point included in each linear regression. Entrance into stationary-phase was defined as the time point at which the growth rate begins to monotonically decrease toward zero and the R2 from the regression was less than 0.98 (indicated by arrows). Data points reflect the means of at least 3 biological replicates. Error bars indicate standard errors of the means. (PDF) [file pgen.1011840.s005.pdf]

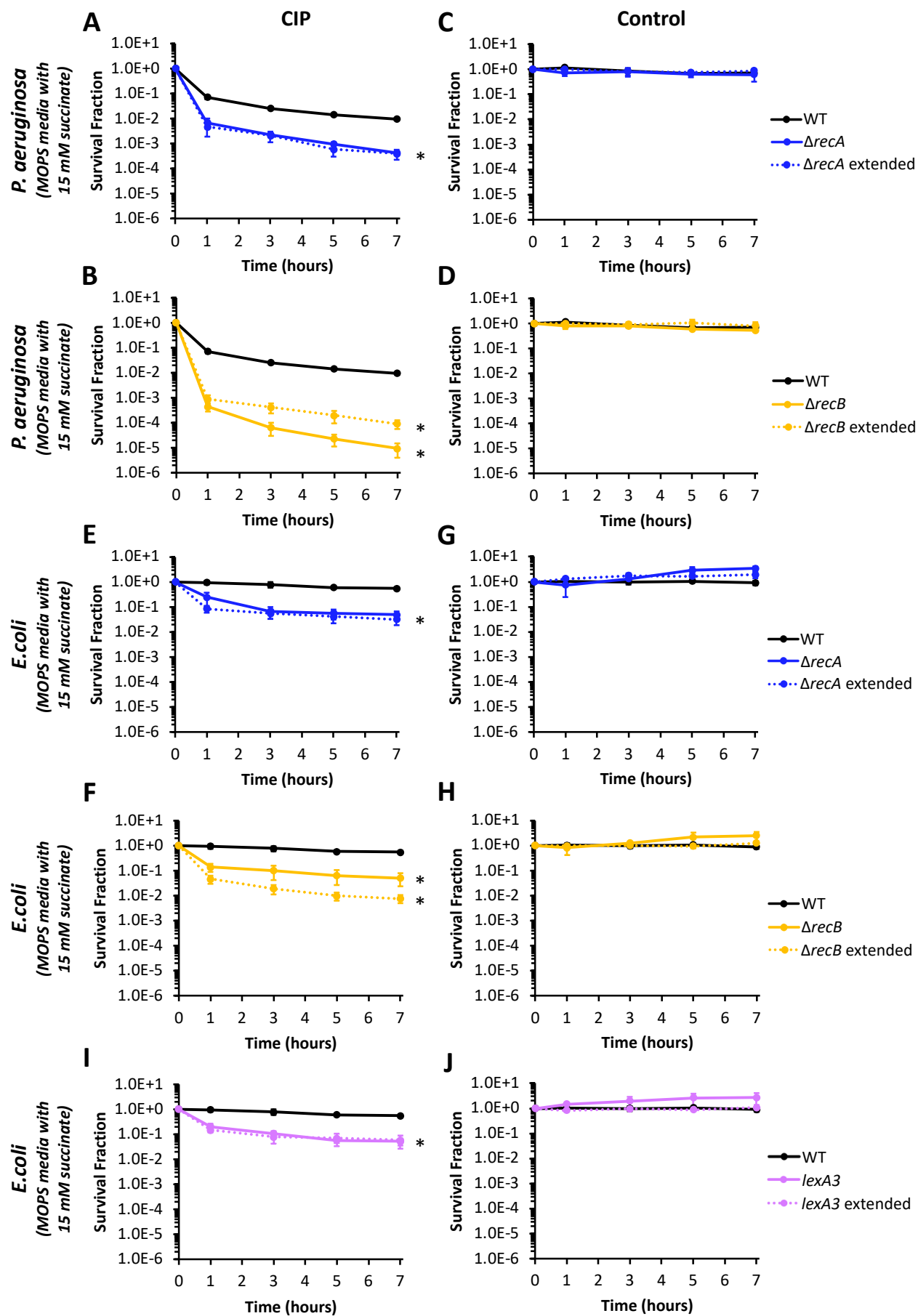

S5 Figure

Supplement: S5 Fig — (A-D) P. aeruginosa PAO1 (A and C) ΔrecA or (B and D) ΔrecB were grown to stationary-phase in MOPS minimal media with succinate for 24 hours (standard incubation) or for an extended period (25 and 29 hours, respectively) to match the time in stationary-phase between WT and mutants (WT was grown for 24 hours). (E-J) E. coli MG1655 (E and G) ΔrecA, (F and H) ΔrecB, or (I and J) lexA3 were grown to stationary-phase in MOPS minimal media with succinate for 28 hours (standard incubation) or for an extended period (29, 30, and 29 hours, respectively) to match the time in stationary-phase between WT and mutants (WT was grown for 28 hours). After the specified incubations, cultures were treated with (A, B, E, F, and I) 10 μg/mL CIP or (C, D, G, H, and J) solvent control. Samples were taken at the indicated time points, washed, and plated on LB agar for CFU enumeration. Data points reflect the means of at least 3 biological replicates. Error bars indicate standard errors of the means. One-way ANOVA with post-hoc Tukey tests were performed on log-transformed survival fractions after 7 hours of CIP treatment to assess significance. *Asterisks denote statistical significance (p ≤ 0.05). (A, E, and I) Survival fractions for each strain with standard and extended incubation were significantly different from those of WT but not each other. (B and F) Survival fractions of ΔrecB with standard and extended incubations were significantly different from those of WT and from each other. (PDF) [file pgen.1011840.s006.pdf]

*P. aeruginosa* (MOPS media with 15 mM succinate)

**A**

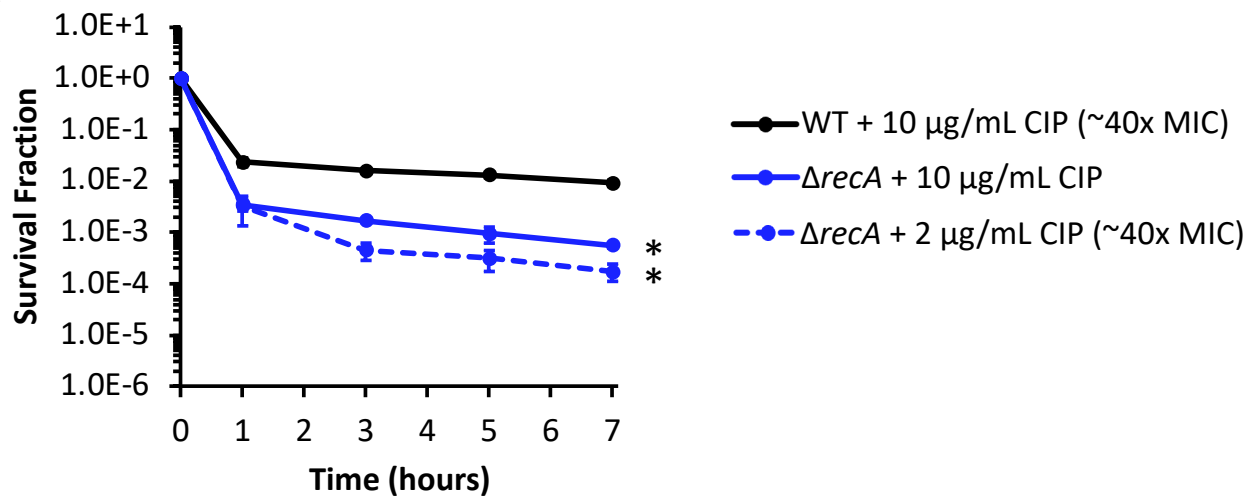

**B**

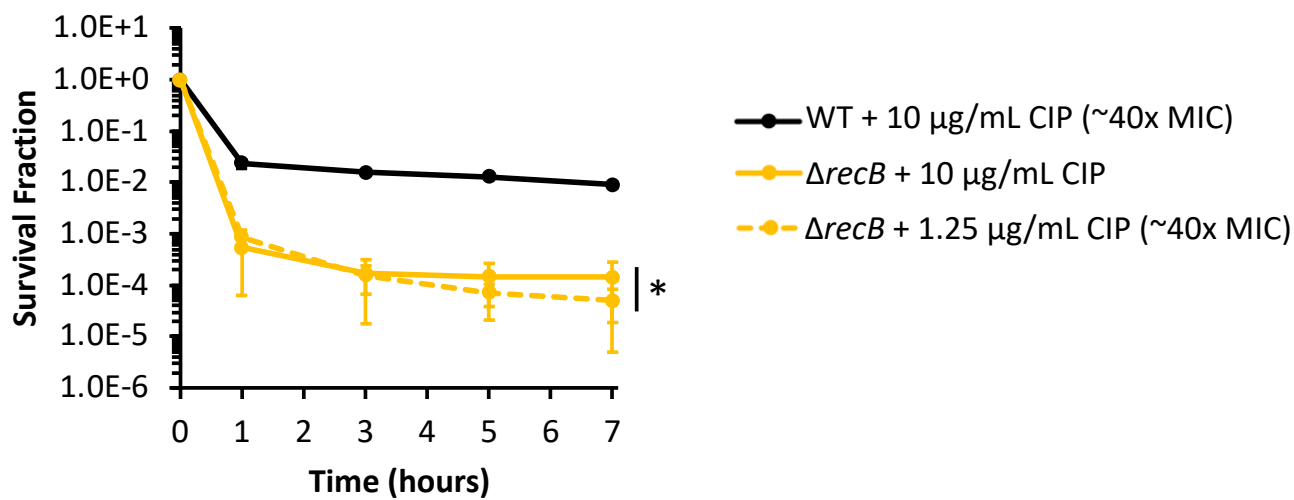

**C**

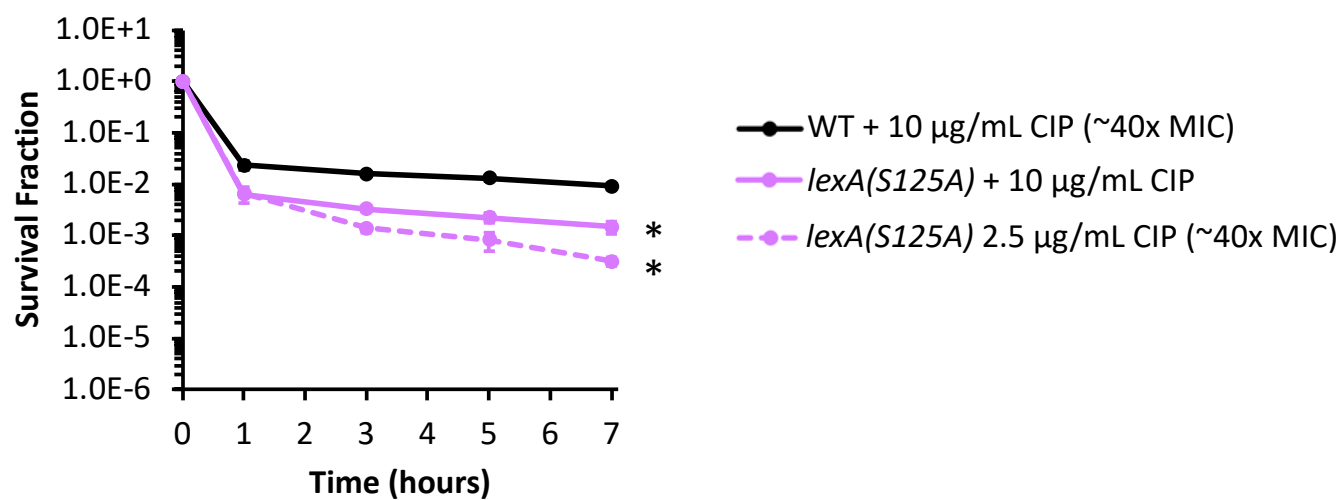

S6 Figure

Supplement: S6 Fig — P. aeruginosa PAO1 WT and mutants with reduced MICs compared to WT were grown to stationary-phase (24 h) in MOPS minimal media with succinate and then treated with 10 μg/mL CIP (40-fold MIC of WT, see S1 Table). During each replicate, each mutant was also treated with CIP at 40-fold MIC. (A) WT and ΔrecA were treated with 10 μg/mL CIP, and ΔrecA was also treated with 2 μg/mL CIP. (B) WT and ΔrecB were treated with 10 μg/mL CIP, and ΔrecB was also treated with 1.25 μg/mL CIP. (C) WT and lexA(S125A) were treated with 10 μg/mL CIP, and lexA(S125A) was also treated with 2.5 μg/mL CIP. Samples were taken at the indicated time points, washed, and plated on LB agar for CFU enumeration. Data points reflect the means of 3 biological replicates. Error bars indicate standard errors of the means. One-way ANOVA with post-hoc Tukey tests were performed on log-transformed survival fractions after 7 hours of treatment to assess significance. *Asterisks denote statistical significance (p ≤ 0.05), where (A) survival fractions of WT, ΔrecA + 10 μg/mL CIP, and ΔrecA + 2 μg/mL CIP are all significantly different from each other; (B) survival fractions of ΔrecB + 10 μg/mL CIP and ΔrecB + 1.25 μg/mL CIP are significantly different from those of WT but not each other; and (C) survival fractions of WT, lexA(S125A) + 10 μg/mL CIP, and lexA(S125A) + 2.5 μg/mL CIP are all significantly different from each other. (PDF) [file pgen.1011840.s007.pdf]

*E. coli* (MOPS media with 10 mM glucose)

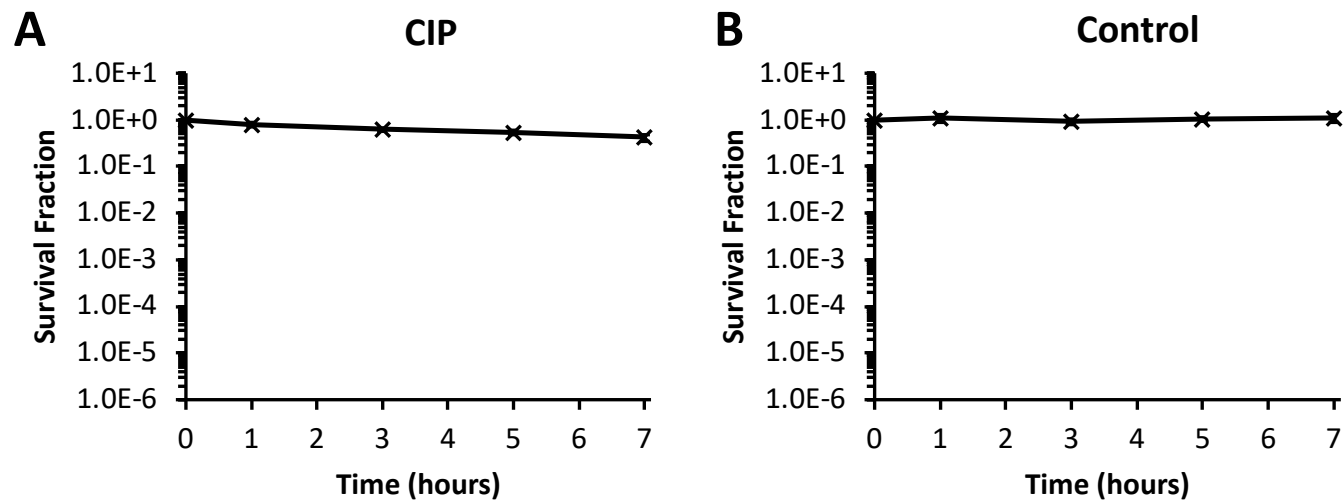

S7 Figure

Supplement: S7 Fig — E. coli MG1655 WT was grown in MOPS minimal media with glucose for 26 hours and then treated with (A) 10 μg/mL CIP or (B) solvent control. Immediately before treatment (t = 0 h) and at t = 1, 3, 5, and 7 h, samples were taken, washed, and plated on LB agar. Data points reflect the means of 3 biological replicates. Error bars indicate standard errors of the means. (PDF) [file pgen.1011840.s008.pdf]

**A** WT

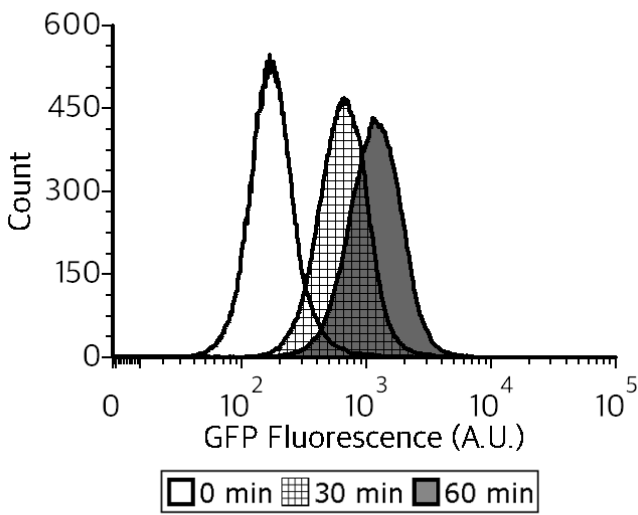

**B** *lexA(S125A)*

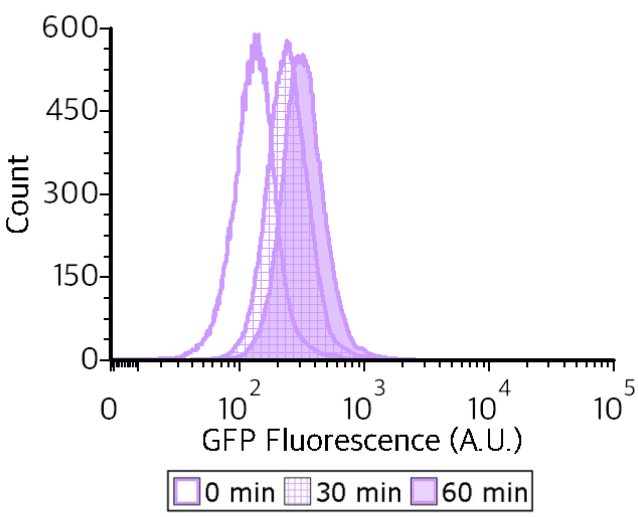

**C**

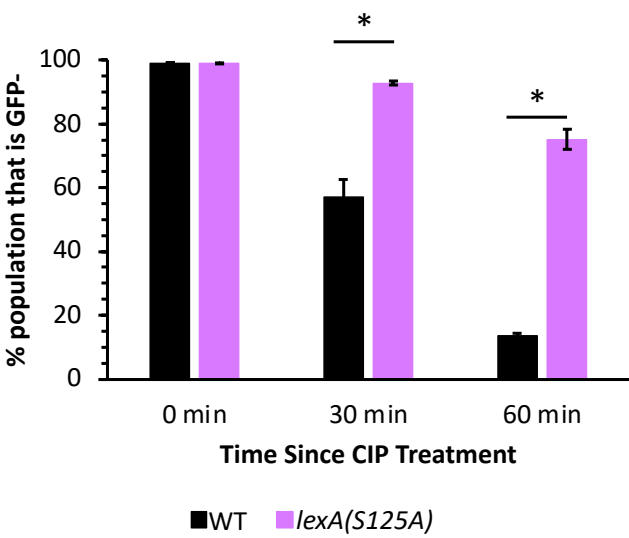

**S8 Figure**

Supplement: S8 Fig — P. aeruginosa PAO1 (A) WT and (B) lexA(S125A) expressing pGL15 (SOS reporter) were grown in LB to exponential-phase and then treated with 10 μg/mL CIP. Immediately before treatment and then after 30 minutes and 60 minutes, samples were removed and fixed. (A and B) Fluorescence distribution was measured using flow cytometry. Histograms are representative of 3 biological replicates. (C) The percentage of the population that was GFP negative at each time point was quantified by gating 99% of the population at t = 0 min and identifying the percentage of the population within this gate at each subsequent time point from the same experiment for each strain. Data reflect the means of 3 biological replicates. Error bars indicate standard errors of the means. Two-tailed t-tests with unequal variances were performed to compare the percentages of the populations of WT and lexA(S125A) that were GFP negative at 30 and 60 minutes. *Asterisks denote statistical significance (p ≤ 0.05). (PDF) [file pgen.1011840.s009.pdf]

*P. aeruginosa* (MOPS media with 15 mM succinate)

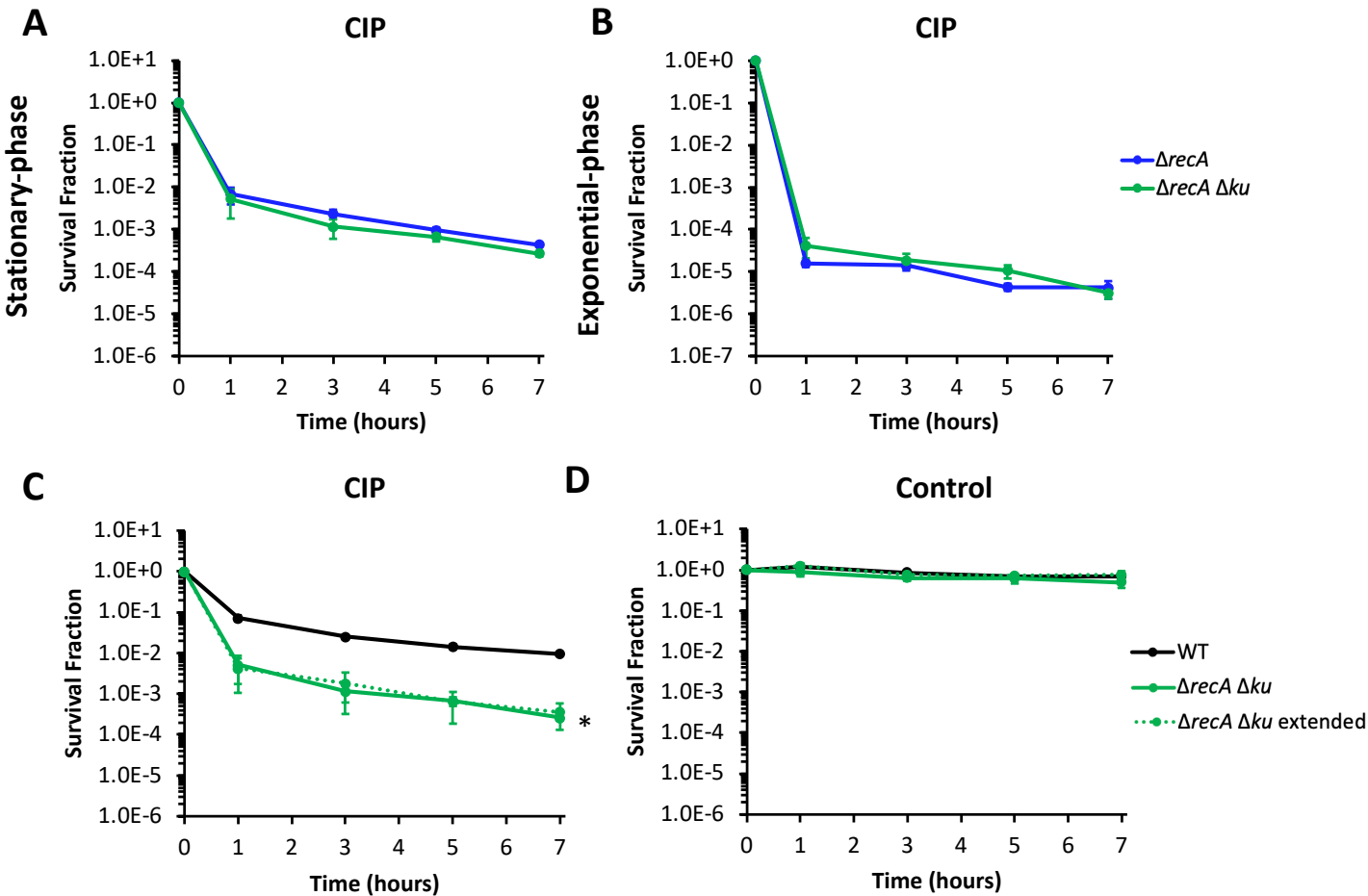

S9 Figure

Supplement: S9 Fig — (A and B) P. aeruginosa PAO1 ΔrecA and ΔrecA Δku were grown to (A) stationary-phase (24 h) or (B) OD600 ~ 0.2 in MOPS minimal media with succinate and then treated with 10 μg/mL CIP. (C and D) P. aeruginosa PAO1 WT and ΔrecA Δku were grown for 24 hours or for an extended incubation of 25 hours (labeled ΔrecA Δku extended) to match the time of ΔrecA Δku cultures in stationary-phase to that of WT prior to treatment. After the specified incubation, cultures were treated with (C) 10 μg/mL CIP or (D) solvent control. Samples were taken at the indicated time points, washed, and plated on LB agar to quantify survivors. Data points reflect the means of at least 3 biological replicates. Error bars indicate standard errors of the means. (A and B) Two-tailed t-tests with unequal variances were performed on log-transformed survival fractions after 7 hours of treatment to assess significance. No statistical significance was found. (C) One-way ANOVA with post-hoc Tukey tests were performed on log-transformed survival fractions after 7 hours of treatment to assess significance. *Asterisk denotes statistical significance (p ≤ 0.05) with respect to WT. (PDF) [file pgen.1011840.s010.pdf]

**A**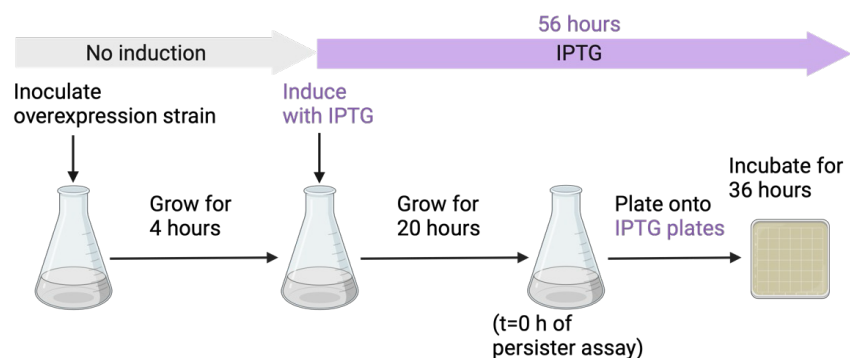**B**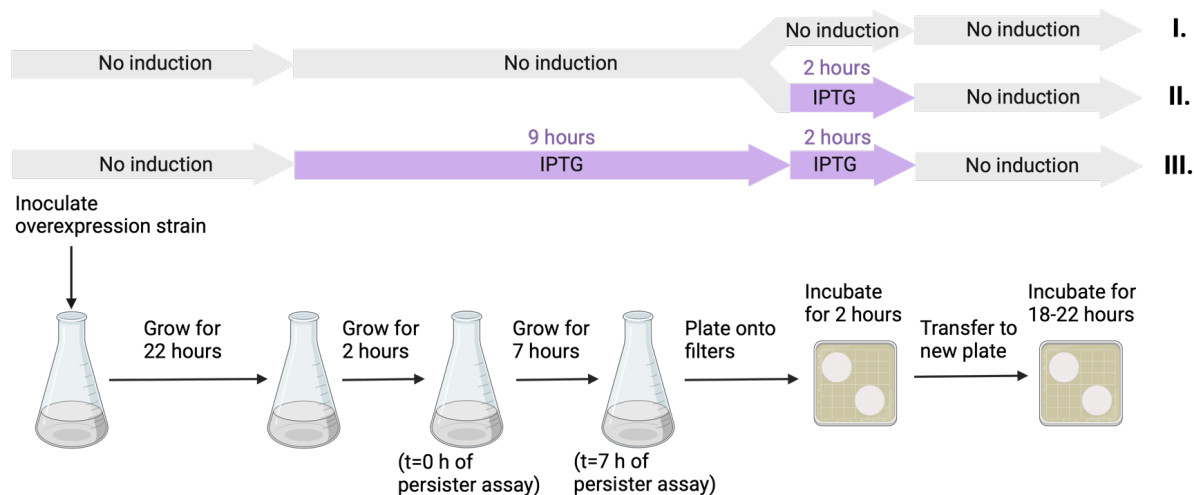**C**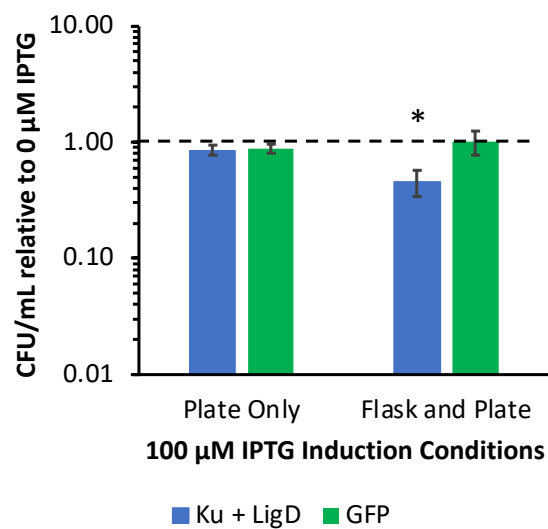**S11 Figure**

Supplement: S11 Fig — (A and B) Schematics demonstrating different methods of inducing Ku and LigD expression in P. aeruginosa. (A) P. aeruginosa PAO1 WT harboring pGL11 or pGL12 were grown for 4 hours in MOPS minimal media with succinate and then induced with IPTG at a range of concentrations. Cultures were incubated for an additional 20 hours, and then samples were removed, washed, and plated on LB agar plates containing IPTG at the same concentration as the flask cultures. Created in BioRender. Leon, G. (2025) https://BioRender.com/y74h037 (B) P. aeruginosa PAO1 WT harboring pGL11 or pGL12 were grown for 24 hours in MOPS minimal media with succinate. Cultures were incubated for an additional 7 hours (corresponds to the time of persister assays) without treatment, and then samples were removed, washed, and plated on filters on LB agar plates. After two hours of incubation, filters were transferred to a second set of LB agar plates and incubated for an additional 18–22 hours. For method I, no IPTG was included to serve as a basis for comparison. For method II, 100 μM IPTG was included only in the first set of LB agar plates. For method III, after 22 hours of initial growth, 100 μM IPTG was added to the cultures. After an additional 9 hours of incubation, samples were plated onto filters on the first set of LB agar plates, which contained 100 μM IPTG. The filters were later transferred to IPTG-free LB agar plates. Created in BioRender. Leon, G. (2025) https://BioRender.com/f26j739 (C) The protocol described in (B) was performed, and culturability was measured as CFU/mL for each induction condition (IPTG on first plate only or IPTG in flask culture and on first plate) relative to CFU/mL with no induction for the same strain during the same experiment. Data reflect the means of at least 3 biological replicates. Error bars indicate standard errors of the means. Two-tailed t-tests with unequal variances were performed on log-transformed relative CFU/mL to assess significance. *Asterisk denot [file pgen.1011840.s012.pdf]

*P. aeruginosa* (MOPS media with 15 mM succinate)

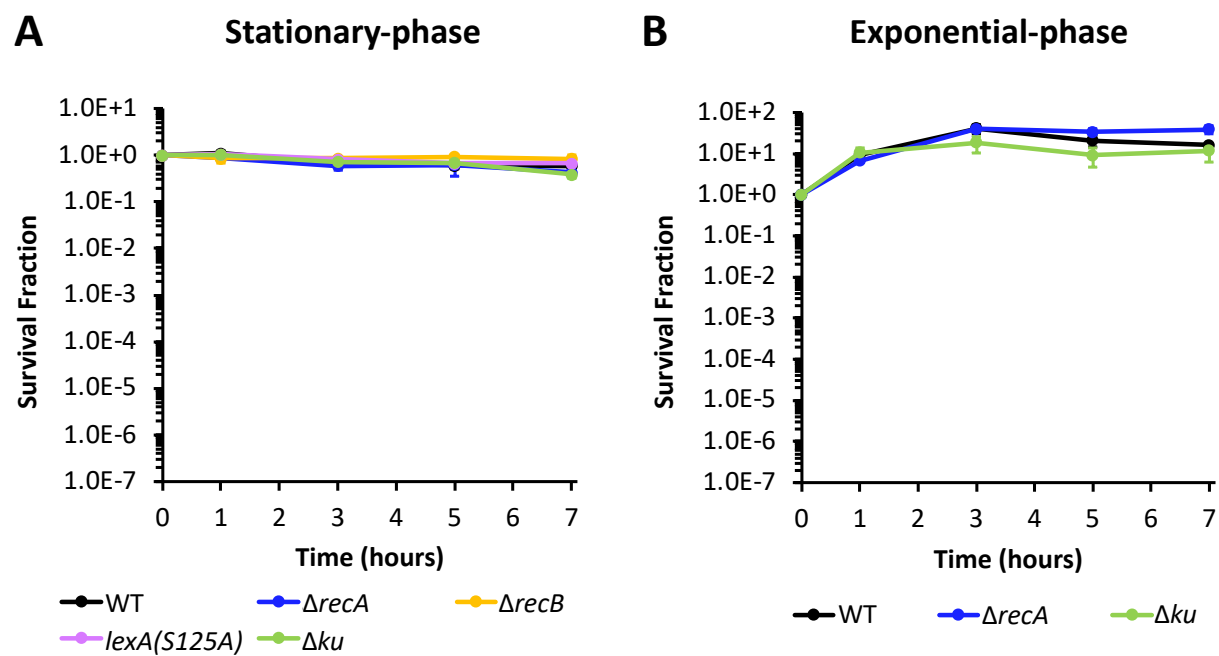

S12 Figure

Supplement: S12 Fig — P. aeruginosa PAO1 strains were grown in MOPS minimal media with succinate to (A) stationary-phase or (B) OD600 ~ 0.2 and then treated with the solvent used to prepare LEV. Samples were taken at the indicated time points, washed, and plated on LB agar for CFU enumeration. Data points reflect the means of at least 2 biological replicates. Error bars indicate standard errors of the means. (PDF) [file pgen.1011840.s013.pdf]
