# Supplementary material for: Conservation and divergence of ciprofloxacin persister survival mechanisms between Pseudomonas aeruginosa and Escherichia coli
Source: PLoS Genet. 2025 Sep 2;21(9):e1011840. doi: 10.1371/journal.pgen.1011840 (PMC12413089; doi:10.1371/journal.pgen.1011840)
Supplement: S10 Fig — P. aeruginosa PAO1 WT expressing pGL10 (Ptac empty), pGL11 (Ptac-sfgfp), or pGL12 (Ptac-ku-ligD) were grown to exponential-phase in LB and then treated with 1 mM IPTG for 4 hours prior to preparation of protein lysates. (A) SDS-PAGE gel. Lanes 1 and 5: Protein ladder. Lane 2: WT + pGL10 (Ptac empty). Lane 3: WT + pGL11 (Ptac-sfgfp). Lane 4: WT + pGL12 (Ptac-ku-ligD). Red arrows indicate bands suspected to contain LigD (94 kDa) and Ku (32.8 kDa) based on size. (B and C) Samples suspected to contain LigD and Ku based on size from lane 4 were cut and analyzed via mass spectrometry. Protein sequence coverage for (B) LigD and (C) Ku samples were 85% and 91%, respectively. Yellow highlight indicates identified peptides. (PDF) [file pgen.1011840.s011.pdf]

A

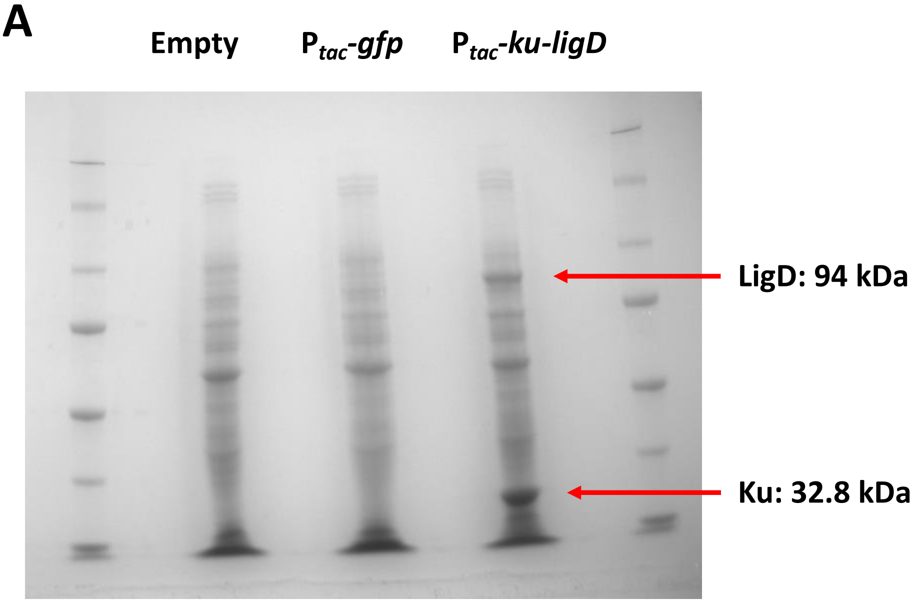

B

LigD: 85% coverage

```
        10         20         30         40         50         60         70
MPSSKPLAEY ARKRDFRQTP EPSGRKPRKD STGLLRYCVQ KHDASRLHYD FRLELDGTLK SWAVPKGPCL
        80         90        100        110        120        130        140
DPAVKRLAVQ VEDHPLDYAD FEGSIPQGHY GAGDVIVWDR GAWTPLDDPR EGLEKGHLSF ALDGEKLSGR
       150        160        170        180        190        200        210
WHLIRTNLRG KQSQWFLVKA KDGEARSLDR FDVLKERPDS VLSERTLLPR HGEAATPAAR PARRGKSGGK
       220        230        240        250        260        270        280
TPMPEWIAPE LASLVEQPPR GEWAYELKLD GYRLMSRIED GHVRLLTRNG HDWTERLPHL EKALAGLGLQ
       290        300        310        320        330        340        350
RSWLDGELVV LDEEGRPDFQ ALQNAFEEGR GENILYVLFD LPYHEGEDLR DVALEERRAR LEALLEGRDE
       360        370        380        390        400        410        420
DPLRFSATLA EDPRDLLASA CKLGLEGVIG KRLGSAYRSR RSNDWIKLKC QLRQEFVIVG YTEPKGSRRH
       430        440        450        460        470        480        490
IGALLLGLYS PDEERRLRYA GKVGSGFTAA SLKKVRERLE PLAVRSSPLA KVPPARETGS VQWVRPQQLC
       500        510        520        530        540        550        560
EVSYAQMTRG GIIRQAVFHG LREDKPAREV TGERPAGPPP LRGARKASAG ASRAATAGVR ISHPQRLIDP
       570        580        590        600        610        620        630
SIQASKLELA EFHARYADLL LRDLRERPVS LVRGPDGIGG ELFFQKHAAR LKIPGIVQLD PALDPGHPPL
       640        650        660        670        680        690        700
LQIRSAEALV GAVQMGSIEF HTWNASLANL ERPDRFVLDL DPDPALPWKR MLEATQLSLT LLDELGLRAF
       710        720        730        740        750        760        770
LKTSGGKGMH LLVPLERRHG WDEVKDFAQA ISQHLARLMP ERFSAVSGPR NRVGKIFVDY LRNSRGASTV
       780        790        800        810        820        830        840
AAYSVRAREG LPVSVPVFRE ELDSLQGANQ WNLRSLPQRL DELAGDDPWA DYAGTRQRIS AAMRRQLGRG
```

C

Ku: 91% coverage

```
        10         20         30         40         50         60         70
MARAIWKGAI SFGLVHIPVS LSAATSSQGI DFDWLDQRSM EPVGYKRVNK VTGKEIEREN IVKGVEYEKG
        80         90        100        110        120        130        140
RYVVLSEEEI RAAHPKSTQT IEIFAFVDSQ EIPLQHFDTP YYLVPDRRGG KVYALLRETL ERTGKVALAN
       150        160        170        180        190        200        210
VVLHTRQHLA LLRPLQDALV LITLRWPSQV RSLDGLELDE SVTEAKLDKR ELEMAKRLVE DMASHWEPDE
       220        230        240        250        260        270        280
YKDSFSDKIM KLVEEKAAKG QLHAVEEEEE VAGKGADIID LTDLLKRSLR SRAGGGKDKG SEKAGADAKG
       290        293
RAKSGASRSR RKA
```

S10 Figure
