# Supplementary material for: Conservation and divergence of ciprofloxacin persister survival mechanisms between Pseudomonas aeruginosa and Escherichia coli
Source: PLoS Genet. 2025 Sep 2;21(9):e1011840. doi: 10.1371/journal.pgen.1011840 (PMC12413089; doi:10.1371/journal.pgen.1011840)
Supplement: S1 Table — CIP and LEV MICs were measured with E-test strips (Liofilchem). MICs represent the range observed for at least 3 biological replicates. (PDF) [file pgen.1011840.s014.pdf]

S1 Table

**Strain**	**MIC (µg/mL)**
**CIP**	
P. aeruginosa PAO1 (ATCC 15692)	
WT	0.19-0.25
Δ*recA*	0.032-0.047
Δ*recB*	0.023-0.032
Δ*recA* Δ*ku*	0.032
lexA(S125A)	0.064
Δ*ku*	0.19
Δ*ligD*	0.19-0.25
E. coli MG1655	
WT	0.012-0.016
Δ*recA*	0.003-0.004
Δ*recB*	0.004
lexA3	0.006-0.008
**LEV**	
P. aeruginosa PAO1 (ATCC 15692)	
WT	0.5-1.0
Δ*recA*	0.094-0.19
Δ*recB*	0.094-0.19
lexA(S125A)	0.25-0.38
Δ*ku*	0.5-1.0
