# Supplementary material for: Conservation and divergence of ciprofloxacin persister survival mechanisms between Pseudomonas aeruginosa and Escherichia coli
Source: PLoS Genet. 2025 Sep 2;21(9):e1011840. doi: 10.1371/journal.pgen.1011840 (PMC12413089; doi:10.1371/journal.pgen.1011840)
Supplement: S2 Table — (PDF) [file pgen.1011840.s015.pdf]

S2 Table

Strain/plasmid	Relevant genotype	Source
	***P. aeruginosa* strains**	
PAO1 WT	PAO1	*Pseudomonas aeruginosa* (Schroeter) Migula (ATCC 15692)
MRSN 1612 WT	MRSN 1612	BEI Resources, NIAID, NIH, *Pseudomonas aeruginosa* , Strain MRSN 1612, NR-51526
Δ*recA*	PAO1 Δ*recA*	Generated using λ Red recombination in PAO1 WT and cured of *gmR* via Flp recombination.
Δ*recB*	PAO1 Δ*recB*	Generated using λ Red recombination in PAO1 WT and cured of *gmR* via Flp recombination.
Δ*ku*	PAO1 Δ*ku*	Generated using two-step allelic exchange in PAO1 WT background.
Δ*ligD*	PAO1 Δ*ligD*	Generated using two-step allelic exchange in PAO1 WT background.
lexA(S125A)	PAO1 *lexA(S125A)*	Generated using two-step allelic exchange in PAO1 WT background.
Δ*recA* Δ*ku*	PAO1 Δ*recA* Δ*ku*	Generated using λ Red recombination in PAO1 Δ*ku* and cured of *gmR* via Flp recombination.
Δ*recA* P_{recA}-empty	PAO1 Δ*recA* *att*Tn7::*gmR*- P_{recA}	Generated using Tn7 transposition to insert *gmR*-P_{recA} cassette into *att*Tn7 site in PAO1 Δ*recA* .
Δ*recA* P_{recA}-*recA*	PAO1 Δ*recA* *att*Tn7::*gmR*- P_{recA}-*recA*	Generated using Tn7 transposition to insert *gmR*-P_{recA}-*recA* cassette into *att*Tn7 site in PAO1 Δ*recA* .
Δ*recB* P_{recB}-empty	PAO1 Δ*recB* *att*Tn7::*gmR*- P_{recB}	Generated using Tn7 transposition to insert *gmR*-P_{recB} cassette into *att*Tn7 site in PAO1 Δ*recB* .
Δ*recB* P_{recB}-*recB*	PAO1 Δ*recB* *att*Tn7::*gmR*- P_{recB}-*recB*	Generated using Tn7 transposition to insert *gmR*-P_{recB}-*recB* cassette into *att*Tn7 site in PAO1 Δ*recB* .
MRSN 1612 Δ*recA*	MRSN 1612 Δ*recA*	Generated using two-step allelic exchange in MRSN 1612 WT background.
	***E. coli* strains**	
MG1655	F-, λ-, *ilvG* -, *rfb-50* , *rph-1*	(7)
Δ*recA*	MG1655 Δ*recA*	(8)
Δ*recB*	MG1655 Δ*recB*	(8)
lexA3	MG1655 *lexA3*	(8)
S17-1	for mating	(9)
	**Plasmids**	
pQE-80L	Vector, ColE1 ori, *ampR* , *lacI*	Qiagen, Valencia, CA
pAS03	Vector, R6K ori, *FRT(minimal)-gmR-FRT*	(10)
pBBR1MCS-2	Vector, pBBR1 oriV, *kanR*	(11)
pEXG2	Vector, ColE1 ori, gmR, *sacB* , *mob*	(12)
pUC18-mini-Tn7T-LAC	Vector, ColE1 ori, *ampR* , *FRT*-*gmR-FRT* and *tac* expression cassette between Tn7 left and right sites	(2)
pTNS2	Vector, R6K γ ori, *tnsABCD*	(2)
pUA66-*lacIq* -P_{T5}-*sfgfp*	Vector, pSC101 ori, *kanR,* P_{T5}-*sfgfp* , *lacIq*	(13)
pUCP18-RedS	Vector, ColE1 ori, pRO1600 oriV, *bla, sacB, araC* , P_{araBAD}-*gam-beta-exo*	(14)
pCP20	Vector, pSC101 ori, *ampR* , *flp*	(15)
pMMB67EH	Vector, RSF1010 oriV, *ampR, lacIq* , P_{tac}	(16)
pGL01	pQE-80L *FRT*	Generated using restriction digestion to insert full-length *FRT* amplified from pAS03 using primer pair 1 into pQE-80L.

pGL02	pQE-80L *FRT-gmR-FRT*	Generated using restriction digestion to insert *gmR -FRT* amplified from pAS03 using primer pair 2 into pGL01.
pGL03	pMMB67EH P_{tac}-*flp*	Generated using Gibson assembly to insert *flp* from pCP20 into pMMB67EH using primer pairs 3 and 4.
pGL04	pGL03 *sacB*	Generated using Gibson assembly to insert *sacB* from pUCP18-RedS into pGL03 using primer pairs 5 and 6.
pGL05	pEXG2 *ku upstream -ku downstream*	Generated using overlap PCR to assemble the sequences directly upstream and downstream of *ku* amplified from PAO1 gDNA using primer pairs 7, 8, and 9 and restriction digestion to insert the assembled product into pEXG2.
pGL06	pEXG2 *ligD upstream-ligD downstream*	Generated using Gibson assembly to insert the sequences directly upstream and downstream of *ligD* from PAO1 gDNA into pEXG2 using primer pairs 10, 11, and 12.
pGL07	pEXG2 *lexA*	Generated using Gibson assembly to insert ~1000 bp of *lexA* and surrounding regions centered approximately at *lexA(S125)* from PAO1 gDNA into pEXG2 using primer pairs 10 and 13.
pGL08	pEXG2 *lexA(S125A)*	Generated using site-directed mutagenesis to create *lexA(S125A)* mutation in pGL07 using primer pair 14.
pGL09	pBBR1MCS-2 with *gmR* replacing *kanR*	Generated using Gibson assembly to replace *kanR* in pBBR1MCS-2 with *gmR* from pEXG2 using primer pairs 19 and 20.
pGL10	pGL09 P_{tac}, *lacIq*	Generated using Gibson assembly to insert P_{tac} expression cassette including *lacIq* from pMMB67EH into pGL09 using primer pair 21.
pGL11	pGL09 P_{tac}-*sfgfp* , *lacIq*	Generated using Gibson assembly to insert *sfgfp* from pUA66-*lacIq* -P_{T5}-*sfgfp* into pGL10 using primer pair 22.
pGL12	pGL09 P_{tac}-*ku-ligD* , *lacIq*	Generated using Gibson assembly to insert *ku* and *ligD* including native RBSs from PAO1 gDNA into pGL10 using primer pairs 23 and 24.
pGL13	pBBR1MCS-2 *sfgfp*	Generated using Gibson assembly to insert *sfgfp* from pUA66-*lacIq* -P_{T5}-*sfgfp* into pBBR1MCS-2 using primer pairs 15 and 16.
pGL14	pBBR1MCS-2 P_{lexA}-*sfgfp*	Generated using Gibson assembly to insert *lexA* promoter from PAO1 gDNA into pGL13 using primer pairs 17 and 18.
pGL15	pGL09 P_{lexA}-*sfgfp*	Generated using Gibson assembly to replace *kanR* in pGL14 with *gmR* from pEXG2 using primer pairs 19 and 20.
pGL16	pUC18-mini-Tn7T-LAC P_{recA} replacing *tac* expression cassette	Generated using Gibson assembly to replace P_{tac} expression cassette in pUC18-mini-Tn7T-LAC with *recA* promoter from PAO1 gDNA using primer pair 25.
pGL17	pUC18-mini-Tn7T-LAC P_{recA}-*recA* replacing tac expression cassette	Generated using Gibson assembly to replace P_{tac} expression cassette in pUC18-mini-Tn7T-LAC with *recA* expressed from native promoter from PAO1 gDNA using primer pair 26.
pGL18	pUC18-mini-Tn7T-LAC P_{recB} replacing tac expression cassette	Generated using Gibson assembly to replace P_{tac} expression cassette in pUC18-mini-Tn7T-LAC with *recB* promoter from PAO1 gDNA using primer pair 27.
pGL19	pUC18-mini-Tn7T-LAC P_{recB}-*recB* replacing tac expression cassette	Generated using Gibson assembly to replace P_{tac} expression cassette in pUC18-mini-Tn7T-LAC with *recB* expressed from native promoter from PAO1 gDNA using primer pairs 28 and 29.
pGL20	pEXG2 MRSN 1612 *recA upstream -recA downstream*	Generated using Gibson assembly to insert the sequences directly upstream and downstream of *recA* from MRSN 1612 gDNA into pEXG2 using primer pairs 30 and 31.
