# Supplementary material for: Conservation and divergence of ciprofloxacin persister survival mechanisms between Pseudomonas aeruginosa and Escherichia coli
Source: PLoS Genet. 2025 Sep 2;21(9):e1011840. doi: 10.1371/journal.pgen.1011840 (PMC12413089; doi:10.1371/journal.pgen.1011840)
Supplement: S3 Table — (PDF) [file pgen.1011840.s016.pdf]

S3 Table

Primer Pair	Primer Sequence	Description of primer pair
**Plasmid Construction**		
1	GCGCATCTCGAGACCGCAGGGGATCTTGAA	Amplify full-length *FRT* sequence from pAS03 for cloning into pQE-80L via restriction digestion with XhoI and BamHI to generate pGL01.
	GCGCGGGGATCCATTGCAGCATTACACGTCTTGA	
2	GCGCATGGATCCAGAGCGCTTTTGAAGCTCACG	Amplify *gmR -FRT* from pAS03 for cloning into pGL01 via restriction digestion with BamHI and SalI to generate pGL02.
	GCGCGGGTCGACAGCGATTGTGTAGGCTGGAG	
3	GCTGTTTTGGCGGATGAGAG	Amplify pMMB67EH for insertion of *flp* downstream of *tac* promoter via Gibson assembly to generate pGL03.
	AGCTCGAATTCTGTTTCCTGT	
4	CAGGAAACAGAATTCGAGCTACTAAGGAGGTTGTATGCCACA	Amplify *flp* from pCP20 for insertion into pMMB67EH downstream of *tac* promoter via Gibson assembly to generate pGL03.
	CTCTCATCCGCCAAAACAGCCACTGTTCACGTCGCACCTA	
5	ATCTGATAGAGAAGGGTTTGCTC	Amplify pGL03 for insertion of *sacB* via Gibson assembly to generate pGL04.
	TTATGGCAGAGCAGGGAAAG	
6	CTTTCCCTGCTCTGCCATAAAATGATACCGCGAGATCCAC	Amplify *sacB* with promoter from pUCP18-RedS for insertion into pGL03 via Gibson assembly to generate pGL04.
	CAAACCCTTCTCTATCAGATGGTGATGACGGTGAAAACCT	
7	GCGCATAAGCTTGCACCATTCTCAAGGCAGGT	Amplify ~500 bp upstream of *ku* from PAO1 gDNA for assembly with downstream region via overlap PCR.
	CATGCCGGTTTCCTCCTCTT	
8	GGCGGCACGCTGTAACCATCAAGAGGAGGAAACCGGCATGTGAGGCGAGCCGCTGGGTA	Amplify ~500 bp downstream of *ku* from PAO1 gDNA for assembly with upstream region via overlap PCR.
	GCGCGGAAGCTTTAGCCGGCAAAACGGTCGAT	
9	GCGCATAAGCTTGCACCATTCTCAAGGCAGGT	3-step overlap PCR to assemble the upstream and downstream *ku* regions for insertion into pEXG2 via restriction digestion with HindIII to generate pGL05.
	GCGCGGAAGCTTTAGCCGGCAAAACGGTCGAT	
10	CATTTATGCTTCCGGCTCGT	Amplify pEXG2 for Gibson assembly to generate two-step allelic exchange plasmids pGL06 and pGL07.
	TAAAGCAAGCTTCTGCAGGT	
11	ACGAGCCGGAAGCATAAATGGGGCAATCTGCACGATGAACA	Amplify ~600 bp upstream of *ligD* from PAO1 gDNA for assembly with downstream region and pEXG2 via Gibson assembly to generate pGL06.
	TCCGCCTTTTTCGCGGACGGGGTGGCTCCTCGGTGCTC	
12	CCGTCCGCGAAAAAGGCGG	Amplify ~600 bp downstream of *ligD* from PAO1 gDNA for assembly with upstream region and pEXG2 via Gibson assembly to generate pGL06.
	ACCTGCAGAAGCTTGCTTTAGAGTTGCAGGTCGGGCATGG	
13	ACGAGCCGGAAGCATAAATGAACTTCATCGTGACGCAGGAC	Amplify ~1000 bp of *lexA* from PAO1 gDNA and surrounding regions centered approximately at *lexA(S125)* for insertion into pEXG2 via Gibson assembly to generate pGL07.
	ACCTGCAGAAGCTTGCTTTACAGCGGGCATCCTGTTCCT	
14	GCGCGGCATGGCCATGAAGGACATC	Amplify pGL07 with *lexA(S125A)* mutation in forward (top) primer to generate pGL08 via site-directed mutagenesis (NEB).
	ACGCGCAACAGGTAGTCG	
15	ATGCCTGCCCCTCCCTTTT	Amplify pBBR1MCS-2 for insertion of *sfgfp* via Gibson assembly to generate pGL13.
	GGCGGCATACGCGATCAT	
16	CGCATGATCGCGTATGCCGCCATGAGCAAAGGAGAAGAACTTT	Amplify *sfgfp* from pUA66-*lacIq* -P_{T5}-*sfgfp* for insertion into pBBR1CMS-2 via Gibson assembly to generate pGL13.
	CAAAAGGGAGGGGCAGGCATGCTTCCTTAGCTCCTGAAAA	
17	ATGAGCAAAGGAGAAGAACTTTTCA	Amplify pGL13 for insertion of *lexA* promoter in front *sfgfp* using Gibson Assembly to generate pGL14.
	GGCGGCATACGCGATCAT	
18	GCATGATCGCGTATGCCGCCGGTTTCTCCGCCTGACAA	Amplify *lexA* promoter from PAO1 gDNA for insertion into pGL13 in front of *sfgfp* using Gibson assembly to generate pGL14.
	AGTTCTTCTCCTTTGCTCATGTCGTCGCTCTGTGTTTTTATC	
19	CTTGTCCAGATAGCCCAGTAGC	Amplify pBBR1MCS-2 and pGL14 to replace *kanR* with *gmR* via Gibson assembly to generate pGL09 and pGL15.
	GGTTCGAAATGACCGACCA	
20	TTGGTCGGTCATTTCGAACCCGATCTCGGCTTGAACGAA	Amplify *gmR* from pEXG2 for replacement of *kanR* in pBBR1MCS-2 and pGL14 via Gibson assembly to generate pGL09 and pGL15.
	TACTGGGCTATCTGGACAAGAGAAAATGCCGATTATGGAAGC	
21	TACAACAGTTTTTATGCATGCGAACGCCAGCAAGACGTA	Amplify P_{tac} expression cassette from pMMB67EH for insertion into pGL09 digested with PvuI and SphI via Gibson assembly to generate pGL10.
	CAACTGTTGGGAAGGGCGATGAAACGCAAAAAGGCCATCC	

22	CAATTTCACACAGGAAACAGAAGAGGAGAATTACCATATGAGC<br>CTCATCCGCCAAAACAGCCATTATTTGTAGAGCTCATCCATGC	Amplify *sfgfp* from pUA66-*lacIq* -P_{T5}-sfGFP for insertion into pGL10 digested with EcoRI and HindIII via Gibson assembly to generate pGL11.
23	CAATTTCACACAGGAAACAGAAGAGGAGGAAACCGGCATGGCG<br>TCAGGCCTTGCGCCGCGA	Amplify *ku* with native RBS from PAO1 gDNA for assembly with *ligD* and insertion into pGL10 digested with EcoRI and HindIII via Gibson assembly to generate pGL12.
24	GTTCGCGGCGCAAGGCCTGAGCACCGAGGAGCCCACCATG<br>CTCATCCGCCAAAACAGCCATCAGCCGCGCCCGAGCTG	Amplify *ligD* with upstream intergenic region from PAO1 gDNA for assembly with *ku* and insertion into pGL10 digested with EcoRI and HindIII via Gibson assembly to generate pGL12.
25	GAAGCTAATTCGATCATGCAGAGTCCGACCTGACCGCA<br>TTCGCGAGGTACCGGGCCCATGAAGTCCTCGCGAAGTCAG	Amplify *recA* promoter from PAO1 gDNA for insertion into pUC18-mini-Tn7T-LAC digested with NsiI and HindIII via Gibson assembly to generate pGL16.
26	GAAGCTAATTCGATCATGCAGAGTCCGACCTGACCGCACTG<br>TTCGCGAGGTACCGGGCCCATCAATCGGCTTCGGCGTCA	Amplify *recA* with native promoter from PAO1 gDNA for insertion into pUC18-mini-Tn7T-LAC digested with NsiI and HindIII via Gibson assembly to generate pGL17.
27	GAAGCTAATTCGATCATGCATGGAGCATGGAGTGAATCTC<br>TTCGCGAGGTACCGGGCCCAGATTCTTCCTCTTCCAGGCTG	Amplify *recB* promoter from PAO1 gDNA for insertion into pUC18-mini-Tn7T-LAC digested with NsiI and HindIII via Gibson assembly to generate pGL18.
28	GAAGCTAATTCGATCATGCATGGAGCATGGAGTGAATCTC<br>GGCATCTGCTGCTGGCTCATGATTCTTCCTCTTCCAGGCTG	Amplify *recB* promoter from PAO1 gDNA for assembly with *recB* and insertion into pUC18-mini-Tn7T-LAC digested with NsiI and HindIII via Gibson assembly to generate pGL19.
29	ATGAGCCAGCAGCAGATGCCC<br>TTCGCGAGGTACCGGGCCCATCATCGCTCGGCTCCCGG	Amplify *recB* from PAO1 gDNA for assembly with native *recB* promoter and insertion into pUC18-mini-Tn7T-LAC digested with NsiI and HindIII via Gibson assembly to generate pGL19.
30	GTAAAGCAAGCTTCTGCAGGGAGGCTGGCTACGTGACCTA<br>TGAAGTCCTCGCGAAGTCAG	Amplify ~500 bp upstream of *recA* from MRSN 1612 gDNA for assembly with downstream region and insertion into pEXG2 digested with SalI and EcoRI via Gibson assembly to generate pGL20.
31	CTGACTTCGCGAGGACTTCAGGCCAATGGCGATCGTGCT<br>GGAAATTAATTAAGGTACCGTGGGACTGAAGCTGCCGCTC	Amplify ~500 bp downstream of *recA* from MRSN 1612 gDNA for assembly with upstream region and insertion into pEXG2 digested with SalI and EcoRI via Gibson assembly to generate pGL20.
**Plasmid verification**		
32	GTGCCACCTGACGTCTAAGAA<br>TATCAACAGGAGTCCAAGCTCA	PCR and sequencing of pGL02 to verify insertion of *FRT-gmR-FRT* cassette. Reverse primer (bottom) was also used to sequence pGL01 to verify insertion of *FRT* site.
33	TGCAGGTCGTAAATCACTGC<br>ACGGCGTTTCACTTCTGAGT	PCR and sequencing of pGL03 to verify insertion of *flp* and PCR of pGL11 and pGL12 to verify insertion of sfgfp or of ku and ligD, respectively, into pGL10.
34	TTGATTTCTGAAAGCGACCA<br>CATTTCGGCTGAGGGTAAAA	PCR of pGL04 to verify insertion of *sacB* .
35	TTGCAGACTACGGGCCTAAA<br>CTCGCGTATCGGTGATTCAT	PCR and/or sequencing of pGL05, pGL06, pGL07, pGL08, and pGL20 to verify insertions/mutations in pEXG2 for allelic exchange.
36	CATCGCTTCATCGGTGCT<br>GTAGGCGGTCACGACTTTG	PCR and sequencing of pGL13 to verify insertion of *sfgfp* into pBBR1MCS-2.
37	CATCGCTTCATCGGTGCT<br>AGTCATGCCGTTTCATGTG	PCR and sequencing of pGL14 to confirm insertion of *lexA* promoter in front of *sfgfp* in pGL13.
38	CGAAGAACTCCAGCATGAGA<br>AAGTCCAGCGCCAGAAACA	PCR of pGL09 and pGL15 to verify replacement of *kanR* with *gmR* in pBBR1MCS-2 and pGL14.
39	TTGACCGAAACGGAGGAAT<br>GGTTCGAAATGACCGACCA	PCR of pGL10 to verify insertion of P_{tac} expression cassette in pGL09.
40	AGTACGAAAAGGGCCGCTAC<br>GGTATAGCCGACGATCACGA	PCR check of pGL12 internal to *ku* and *ligD* inserts to verify insertion of both genes next to each other into pGL10 (secondary check in addition to primer pair 33).
41	GGGGTGGAAATGGAGTTTTT<br>CGACCGAAAAGATCAAGAGC	PCR and/or sequencing of pGL16, pGL17, pGL18, and pGL19 to verify insertion of P_{recA}, P_{recA}-*recA*, P_{recB} and P_{recB}-*recB* into pUC18-mini-Tn7T-LAC, respectively.
42	GCCATGCAACTGCTCTACCT	Additional primer for sequencing of pGL19 to verify insertion of P_{recB}-*recB* into pUC18-mini-Tn7T-LAC.

**Mutant construction (PCRs and overlap PCRs) via λ Red Recombination**		
43	TCGGCGAAAGCCTTCGTTAC<br>TGAAGTCCTCGCGAAGTCAG	PCR of ~600 bp upstream of *recA* from PAO1 WT gDNA for assembly with *FRT-gmR-FRT*.
44	GGCCAATGGCGATCGTGCT<br>CCGGAGTTCCGCAACCTGAC	PCR of ~600 bp downstream of *recA* from PAO1 WT gDNA for assembly with *FRT-gmR-FRT.*
45	TTCGTGGCCGTCCGGCCGCCCTGACTTCGCGAGGACTTCAGAGGCCCTTTCGTCTTCA<br>CGCACGGCGACGGGCGTATCGAGCACGATCGCCATTGGCCATTAAGCTTGGCTGCAGGT	PCR amplify *FRT-gmR-FRT* from pGL02 with ~40 nucleotides of homology to *recA* upstream and downstream PCR products.
46	TCGGCGAAAGCCTTCGTTAC<br>CCGGAGTTCCGCAACCTGAC	3-step overlap PCR to assemble the upstream *recA, FRT-gmR-FRT*, and downstream *recA* PCR products for λ Red recombination in WT, or PCR amplify Δ*recA::gmR* from gDNA for λ Red recombination in Δ*ku*.
47	CGACCTGCTCTGGCGCTAC<br>GCTCATGCCTGCTCCTCCG	PCR of ~650 bp upstream of *recB* from PAO1 WT gDNA for assembly with *FRT-gmR-FRT*.
48	CCGGGAGCCGAGCGATGAAT<br>GCGCTGGCCGTCTTCCGTAG	PCR of ~650 bp downstream of *recB* from PAO1 WT gDNA for assembly with *FRT-gmR-FRT*.
49	TGGCTGCCCTGGGACGTGGCGCGGAGGAGCAGGCATGAGCGAGGCCCTTTCGTCTTCA<br>GCCAGTTGCTTGAGGGTCGGATTCATCGCTCGGCTCCCGGATTAAGCTTGGCTGCAGGT	PCR amplify *FRT-gmR-FRT* from pGL02 with ~40 nucleotides of homology to *recB* upstream and downstream PCR products.
50	CGACCTGCTCTGGCGCTAC<br>GCGCTGGCCGTCTTCCGTAG	3-step overlap PCR to assemble the upstream *recB, FRT-gmR-FRT*, and downstream *recB* PCR products for λ Red recombination in WT.
**Strain verification (genetic deletion mutants and Tn7 insertion)**		
51	CAAGACCACCCTGACCCTCT<br>CCAGTACCGAACCGATTTCC	PCR amplification internal to *recA* to verify loss of *recA* gene in PAO1 and MRSN 1612.
52	GTCCCTTTCCGTTTCTACCC<br>CGACCGAAAAGATCAAGAGC	PCR amplification of *recA::gmR* with forward primer external to *recA* and reverse primer internal to *gmR*.
53	GTCCCTTTCCGTTTCTACCC<br>AAAGGCACTTTCGTGATTCG	PCR amplification and/or sequencing external to *recA* in PAO1.
54	GTTCACCCAGAACCTGGAAA<br>GCGCAGGTAGGTATGGATGT	PCR amplification internal to *recB* to verify loss of *recB* gene.
55	CTCGGGTCTATCCGGACTTC<br>CGACCGAAAAGATCAAGAGC	PCR amplification *recB::gmR* with forward primer external to *recB* and reverse primer internal to *gmR*.
56	TCGAGCGGGACTGGGATG<br>GTTGCGCTACCTGGTCGT	PCR amplification and/or sequencing external to *recB.*
57	AGTACGAAAAGGGCCGCTAC<br>ACCTCCTCCTCTTCCTCCAC	PCR amplification internal to *ku* to verify loss of *ku* gene.
58	CTGAGCCGGGTGATCCAG<br>GCGCAGCTCCAGGTCCAG	PCR amplification and sequencing external to *ku.*
59	AAGCAGTCCCAGTGGTTCCT<br>GGTATAGCCGACGATCACGA	PCR amplification internal to *ligD* to verify loss of *ligD* gene.
60	GCTCCGGGATCGGATACT<br>GTCAGGTTGAAACGCTCCAG	PCR amplification and sequencing external to *ligD.*
61	GATCAGACGCAACGAGGTTT<br>GCCTTCGATGATCAGTTCCT	PCR amplification external to *lexA*, forward primer (top) also used for sequencing.
62	GACATGGTGCTGTTCTACGG<br>CAACAGGCTGGAAAGGTTGT	PCR amplification of *phzM* gene from PAO1 and MRSN 1612 to verify all colonies are *P. aeruginosa* and not *E. coli* (17).
63	ACTGGGCCTTTCGTTTTATC<br>CTGTGGGTATTGGGCATCG	PCR amplification of Tn7 insert with forward primer internal to insert and reverse primer specific to PAO1 genome to verify insertion of complementation empty control cassettes (P_{recA} or P_{recB}) at *attTn7* site.
64	CAAGACCACCCTGACCCTCT<br>CTGTGGGTATTGGGCATCG	PCR amplification of Tn7 insert with forward primer internal to *recA* and reverse primer specific to PAO1 genome to verify insertion of complementation cassette at *attTn7* site.
65	GATCTTCGAGCGCATCTACC<br>CTGTGGGTATTGGGCATCG	PCR amplification of Tn7 insert with forward primer internal to *recB* and reverse primer specific to PAO1 genome to verify insertion of complementation cassette at *attTn7* site.
66	GTCCCTTTCCGTTTCTACCC<br>GCTGGCTGAAAGGCACTTTC	PCR amplification external to *recA* in MRSN 1612.
